# Advancing biomonitoring of eDNA studies with the *Anaconda* R package: Integrating soil and One Health perspectives in the face of evolving traditional agriculture practices

**DOI:** 10.1371/journal.pone.0311986

**Published:** 2025-01-16

**Authors:** Pierre-Louis Stenger, Audrey Léopold, Kelly Dinh, Pierre Mournet, Nadia Robert, Julien Drouin, Jacques Wamejonengo, Sylvie Russet, Thomas Ibanez, Laurent Maggia, Fabian Carriconde

**Affiliations:** 1 Équipe ‘ Sol & Végétation’ (SolVeg), Institut Agronomique néo-Calédonien (IAC), Nouméa, New Caledonia; 2 UMR AGAP Institut, Université Montpellier, CIRAD, INRAE, Institut Agro, Montpellier, France; 3 CIRAD, UMR AGAP Institut, Montpellier, France; 4 AMAP, Université Montpellier, CIRAD, CNRS, INRAE, IRD, Montpellier, France; 5 CIRAD, UMR AGAP Institut, Nouméa, Nouvelle-Calédonie, France; 6 Institute for Exact and Applied Sciences, Université de la Nouvelle-Calédonie, Nouméa, New Caledonia; ICAR-Indian Insitute of Soil Science, Bhopal, INDIA

## Abstract

Soil health and One Health are global concerns, necessitating the development of refined indicators for effective monitoring. In response, we present the *Anaconda* R Package, a novel tool designed to enhance the analysis of eDNA data for biomonitoring purposes. Employing a combination of different approaches, this package allows for a comprehensive investigation of species abundance and community composition under diverse conditions. This study applied the *Anaconda* package to examine the impact of two types of duration fire-fallow cropping systems, using natural forests as a reference, on soil fungal and bacterial communities in Maré Island (New Caledonia). Condition-specific taxa were identified, particularly pathogenic fungi and bacteria, demonstrating the importance of long-term fallowing efforts. Notably, this package also revealed the potential contributions of beneficial soil microbes, including saprophytes and plant-endophyte fungi, in suppressing soil-borne pathogens. Over-represented microbial ASVs associated with both plant and animal pathogens, including those of potential concern for human health, were identified. This underscores the importance of maintaining intrinsic balance for effective disease suppression. Importantly, the advanced analytical and statistical methods offered by this package should be harnessed to comprehensively investigate the effects of agricultural practice changes on soil health within the One Health framework. Looking ahead, the application of this method extends beyond the realm of One Health, offering valuable insights into various ecological scenarios. Its versatility holds promise for elucidating complex interactions and dynamics within ecosystems. By leveraging this tool, researchers can explore the broader implications of agricultural practice modifications, facilitating informed decisions and sustainable environmental management.

## Introduction

The One Health concept highlights the close connections and the interdependency between humans, animals, plants and the surrounding environment [1]. Soil health constitutes a keystone element of One Health. Indeed, soils are vital living ecosystems that support ecosystem services and subsequently sustain plants and animals’ health, including humans [2, 3]. Human well-being is intrinsically tied to the soil’s capacity to provide food in quantity and quality [4]. To illustrate this point, it has been estimated that about 95% of our food comes from soils [5]. In addition to supplying nutrients to humans, soils are a reservoir of beneficial and detrimental microorganisms [6–10]. The latter include fungi, bacteria, nematodes and viruses that spend their entire life cycle or part of it in soils; incidental presence can also occur due, for instance, to anthropogenic activities [7]. Though these soil-borne pathogens represent a small fraction of living organisms in soils, they can potentially cause serious human and plant infectious diseases and outbreaks [7, 11–13]. Agricultural practices (e.g., organic amendment, tillage, conservation tillage, crop rotations, fallow period, and use of agrochemical products) can affect soil microbial communities and can either promote or suppress soil pathogens [2, 7, 14, 15]. Thus, as stated by [16] healthy soil should display, by definition, a low pathogens and related diseases level. [2] in their recent review (in accordance with the European Commission’s recommendations [3]), pointed out the lack of biological indicators in soil health assessments and proposed the inclusion of soil biodiversity and pathogens as indicators. The evaluation of pathogens risk is challenging and requires the use of appropriate analytical and statistical methods for the establishment of sensitive, informative and feasible ‘biological indicators’ (also called ‘bioindicators’) [2, 7, 17]. To address this ambitious task, it necessitates the conjunction of diverse disciplines (e.g., agronomy, ecology, bioinformatics, biostatistics, and social science), and a close appropriation of the new emerging technologies for accessing this hidden biodiversity.

Ecosystem monitoring powered by environmental ‘omics’ represents a revolutionary toolbox that is increasingly being used [18]. Among this ‘ecogenomic toolbox’ [19], the taxonomy-based implementation methods rely on environmental DNA (eDNA). The term ‘eDNA’ generally means DNA extracted from an environmental sample without isolating the target organism [20]. The eDNA approach has been applied to diverse environments, from terrestrial to deep-sea habitats, and a large array of organisms, from microscopic to macroscopic forms (e.g., fungi, bacteria, insects, plants and fishes) [21–23]. High‐throughput eDNA amplicon sequencing–metabarcoding of eDNA–has been recently used for estimating environmental quality from the diversity, composition, structure and functioning of biological communities [24–26]. As an example, in the context of ecological restoration of degraded lands, soil microbial phyla and functional groups were newly investigated in different regions and proposed as potential indicators of ecosystem recovery [25, 27, 28]. In addition to this, some community analyses take into consideration the significant variations in relative abundances of taxa at the species level in terms of Operational Taxonomic Units (OTUs) or Amplified Sequence Variants (ASVs)) [27, 29, 30].

To find a relative abundance of species correlated to a condition, a current consensus seems to have been found by the scientific community with the use of the DESeq2 tool [31]—a tool normally used for gene expression (transcriptomics) [29, 30, 32] and not for eDNA metabarcoding studies. However, some limitations appear as there are disparities between studies in the way this tool is used in metabarcoding research. There are many standardisation (normalisation) methods, and they are sometimes used at different stages of analysis. Such as examples, the method of normalisation that is sometimes independent of the rarefaction or not (e.g., [33] *vs*. [34]); the use of rarefied data or not (independently of the normalisation, like in [35] *vs*. [36]); the use of DESeq2 normalisation instead of rarefaction (e.g., [37]). Also, the notion of enrichment does not seem to be the same depending on the study (e.g., [30] *vs*. [38]). And lastly, the use of taxonomic rank is not similar between studies (e.g., [32] *vs*. [39]). All this makes it difficult to compare studies. These differences stem from the absence of standardised guidelines or manuals for the use of this kind of tool for metabarcoding studies, which prevents researchers from following a reproducible and validated methodology. In transcriptomics, to compare the relative changes between different conditions in the expression levels of a gene or a protein, the ‘Log-fold change’ measurement is used [31, 40, 41]. It is calculated as the logarithm of the ratio of the values. A significant positive log-fold change indicates an enrichment (a greater relative abundance), whereas a negative log-fold change indicates a depletion (a lower relative abundance). Since these statistics are originally used for genomics/transcriptomics, a genetic enrichment *stricto sensu* corresponds to a group of genes that have a similar biological function and are expressed in the same way and there is therefore genetic enrichment for a given function [42]. In the case of taxonomy (by parallelism with genomics), literally, this would correspond more to enrichment by several ASVs or OTUs (and not just one from relative abundance values) that share a higher taxonomic rank (e.g., Kingdom, Class, Order, Family, or Genus–like in [38]), or a similar biological/ecological function (e.g., plant pathogen for example as in FUNGuild [43]).

Here, the *Anaconda* R package [44] was developed with the ideas of homogenising and reframing the metabarcoding analyses using the DESeq2 tool (named ‘targeted’ analysis)—to address the points on the use of statistics discussed above (LogFoldFC, DESeq2, etc.), and to go further in the analysis of taxonomic enrichment (named ‘global’ analysis). Taxonomic enrichment here, *stricto sensu*, allows highlighting a particular taxonomic rank that is carried by several phylogenetically related species. In the field of identifying bioindicators, working at higher taxonomic ranks than the species can be particularly relevant [25, 45, 46]. Taxonomic enrichment analysis methods can therefore find taxonomic ranks that are condition-specific over- or under-represented. This ‘global’ analysis approach follows [41] methods for gene expression, which used a hierarchical clustering tree of significant Gene Ontology (GO) categories based on shared genes (e.g., Rank-based Gene Ontology Analysis with Adaptive Clustering—RBGOA). This method was adapted in the *Anaconda* R package to the taxonomy, to obtain an enrichment based on taxonomic ranks (*i*.*e*., Kingdom, Class, Order, Family, Genus, and Species). This shift between GO and a taxonomy ontology was possible due to the work of [47] who adapted the GO system to the NCBI Taxon terms. We believe that such a combination of ‘targeted’ and ‘global’ approaches could, in the near future, boost the use of DNA metabarcoding in biomonitoring and could even represent the next breakthrough in the assessment of soil health and One Health.

**Fig 1:**
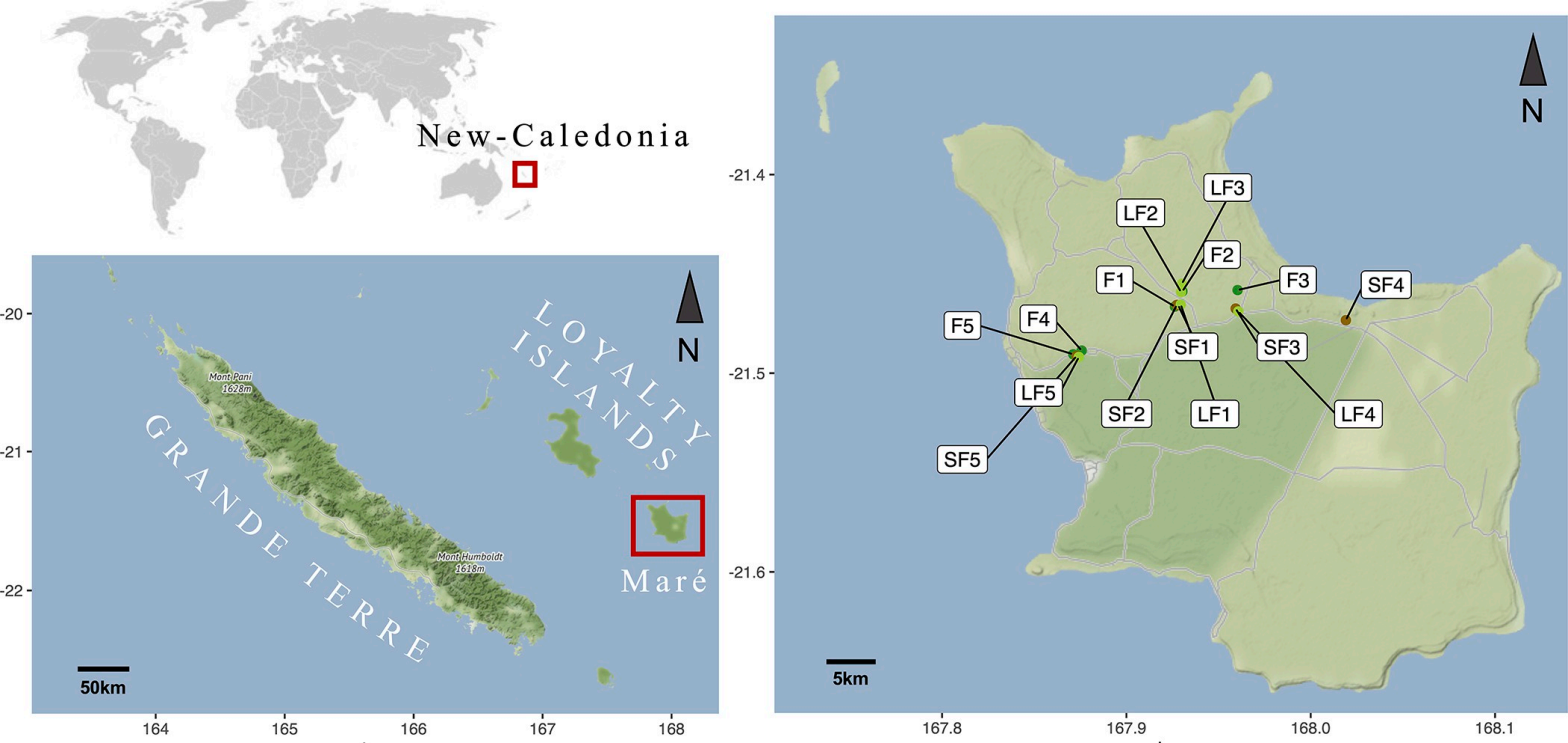
Situation of New Caledonia in the world, and of Maré Island within this archipelago. Location of the different sampling sites in Maré Island. SF is for Short Fallow (brown disk); LF is for Long Fallow (lightgreen disk) and F is for Forest (forest green disk). Map realised with the R package *marmap*.

Analyses were performed on soil fungal and bacterial communities from Maré Island, an island which is part of the Loyalty Island and the French archipelago of New Caledonia (Fig 1). In the Loyalty Islands, indigenous people have traditionally practised fire-fallow agriculture [48]. In Maré, yam cultivation, which displays a high symbolic value, is carried out after low burning (ecoburial) in forests and can be followed, before a fallow period, by vegetable or fruit plantations in the two succeeding years. Societal transformations have led to changes in the traditional agricultural practices on the island [49]. Indeed, fallow periods that last for one to two decades, are more and more frequently limited to a few years ([50]; Drouin, pers. com.).

There is worldwide very limited information on how fallow practices affect soil properties, particularly concerning soil microorganisms [15, 51–55]. The few studies undertaken showed that the effects of the fallow period on microbial diversity are inconsistent, with findings ranging from increases to decreases, or no clear changes [15, 51, 54]. In terms of composition, fallow treatment seems overall to induce changes in fungal and bacterial assemblies [15, 51–54]. More in depth [15], revealed that fallow management in a crop cultivation in China decreased the relative abundance of fungal plant pathogens in soil. However, as stated by the authors, due to the absence of replicated plots (*i*.*e*., only one field plot considered per condition), these results are preliminary. For developing sustainable agricultural practices, it is thus crucial to determine in which extent fallow management influence soil microbial communities, and subsequently soil health.

In this context, our main objective was to determine how changes in traditional agricultural practices in Maré Island impact the soil microbial communities, with both ‘classical’ community analyses and our newly developed methodology implemented in the *Anaconda* R package. We hypothesised that the reduction in the fallowing period would lead to a possible emergence of fungal and bacterial pathogens in soils. To test this hypothesis, plots were established in cultivations differing in their fallowing length, *i*.*e*., short fallow (SF) versus long fallow (LF), and compared to ‘natural’ forests (F) that were used as reference ecosystems. Soil bacterial and fungal communities were assessed using high-throughput amplicon sequencing of environmental DNA (eDNA). In addition, we also looked at other indicators of soil health [3], such as soil organic carbon, soil nutrients content, pH, vegetation cover, and another biological group that corresponds to the nematodes (characterised using a morphological approach). We subsequently determined whether these parameters were related or not to the soil microbial communities, since they are known to be involved in the accumulation or suppression of pathogens.

## Materials and methods

### Experimental design

#### Study sites

The archipelago of New Caledonia is located in the southwestern Pacific, just above the Capricorn tropic, about 1500 km east of Australia and 2000 km north of New Zealand (Fig 1). The New Caledonian archipelago encompass the Loyalty Islands, which includes Maré Island (Fig 1 –map realised with the R package *marmap* V. 1.0.10 [56]). The Maré Island (Fig 1). comprise four main types of soils [57]. Among them, the Gibbsic Ferralsol are known for their extraordinary content of organic matter (humic soils) and gibbsite [49] and used for yam (*Dioscorea* sp.) cultivation. All sampling in this study took place on this type of soil.

#### Conditions and soil sampling

Three condition types were studied: (1) fields that were recently (two to three years ago) cultivated and harvested, then let in fallow, representing the short fallow condition (SF), (2) fields that were last cultivated and harvested ten to twenty years ago and which will be planted in the year of the study, called the long fallow condition (LF), and (3) lands that have never been cultivated and are used as a reference, called the forest condition (F). Five plots of 20 x 20 m were established per condition, totalling 15 plots (Fig 1 and S1 Fig). In each 20 x 20 m plot, four 5 x 5 m sub-plots were placed in the corners and one in the centre. Five soil samples were collected from each sub-plot at a depth of 0–15 cm using a 5 cm diameter auger. The samples from each plot were combined to form a composite soil sample, resulting in 15 composite samples. These samples were sieved on site using 5 mm and 2 mm sieves, placed in a cooler, and stored at 4°C overnight before being flown to Grande Terre (Fig 1). The soil samples were then divided for analysis: one part for DNA extraction (stored at -20°C) at the Plateforme du Vivant in Nouméa, and the other part sent to France within five days for soil organo-physical-chemical analysis and nematode characterisation.

### Soil organo-physico-chemical analyses

All organic-physical-chemical analyses were carried out by an independent laboratory for analysis, study and advice on soil biology (Celesta lab, https://celesta-lab.fr); see methods S1 for more information.

### Plant community inventory

Plots of 20 x 20m were inventoried for plant species with DBH > 5cm. In each plot, four 5 x 5m sub-plots (same as above) were established, where plant species over 1m in height were recorded and measured. Additionally, smaller plant species (less than 1m in height) were counted within these sub-plots.

### Nematodes survey

On the same soil samples used for previous analyses, a survey of nematodes was realised by the independent engineering office Elisol environnement (https://www.elisol.fr). The taxonomic distinction was made up to the families. The abundance per site was also recorded (number of individuals per 100g of dry soil).

### Molecular method

#### Environmental DNA extraction, libraries generation and sequencing

Environmental DNA extraction, libraries generation and sequencing we realized as previously described in [25]. The Regional Genotyping Platform (GPTR Génotypage, https://www.gptr-lr-genotypage.com/) of the UMR AGAP (CIRAD—INRAE—Montpellier SupAgro) performed the libraries generation and sequencing. Approximately 13 million paired reads of 250 bp length were obtained for both ITS2 (Fungi) and V4 (Bacteria) in independent sequencing runs.

### Bioinformatics

#### Working environment

The pipeline was run on the Nouméa Institut de Recherche pour le Développement (IRD) cluster under CentOS Linux release 8.3.2011. Downstream analysis has proceeded on macOS Mojave 10.14.6 (x86_64-apple-darwin17.0 (64-bit)). All scripts created and used for this pipeline can be found at https://github.com/PLStenger/Diversity_in_Mare_yam_crop.

#### Qiime2 framework

Microbiome analysis was performed using the QIIME 2 framework V. 2021.4.0 [58]. Dereplicated and trimmed sequences were imported into the framework as paired-end (Phred33V2) sequences and denoised using the DADA2 plugin, based on the DADA2 V. 1.8 R library [59], which removed singletons, chimaeras, and sequencing errors and processed the sequences into a table of exact amplicon sequence variants (ASVs) [60]. Negative control library sequences were used as in [61]. ASVs that were present in only a single sample were filtered, based on the idea that these may not represent real biological diversity but rather PCR or sequencing errors. Finally, all samples were rarefied to the sample with the lowest number of reads, to keep at the higher number of samples (S2 and S3 Figs).

### Statistical analyses

#### Soil microbial diversity, composition and structure

Statistical analyses were performed using the R software environment V. 4.2.1 [62]. For diversity, the observed number of ASVs [63], Chao1 [64], Simpson evenness [65] Pielou evenness [66], Shannon entropy [67], and Faith PD [68] were performed using Kruskal-Wallis test after checking the normal law by Shapiro test. Bray-Curtis dissimilarity [69] and Jaccard similarity index [70] matrices were calculated with the *q2-diversity* tool. These statistics and their significance post hoc test were obtained with the *agricolae* R package V. 1.3–5 [71]. Boxplots were realised with *ggplot2* R package V. 3.3.5 [72] (S4 and S5 Figs). For fungal functional assignments, we follow the method implemented in [25]. For bacterial functional traits assignment, the database from [73] was used.

As Archaea becoming a growing kingdom that is studied with the V4 markers in soil analysis [74–76] and as there is a unique founded Phyla (Crenarchaeota) and a unique Class (Nitrososphaeria) in our dataset, we included them in our bacteria analysis in the composition bar plots. For all Phyla (ITS2 and V4), Kruskal-Wallis tests were performed on the proportion of the relative abundances between conditions (e.g., SF *vs*. LF *vs*. F).

Regarding soil microbial community structure analyses, distance matrices based on the Bray-Curtis measurement were visualised using non-metric multidimensional scaling (NMDS) with *vegan* R package V. 2.5–7 [77] and *ggplot2* R package V. 3.3.5 [72]. Differences between microbial communities were tested using PERMANOVA, with 9999 permutations with *vegan* R package V. 2.5–7 [77] and the post hoc test was realised with the *pairwiseAdonis* R package V. 0.4 [78].

#### Relationships between soil chemical properties, plants and nematodes on microbial communities

All organic-physical-chemical, plants and nematodes differences between conditions (e.g., F *vs*. LF; F *vs*. SF; LF *vs*. SF) were checked previously using Kruskal-Wallis tests. A soil texture triangle (S6 Fig) was realised with the *ggplot2* R package V. 3.3.5 [72]. We examined relationships between soil fungal and bacterial communities, soil chemical properties, plant and nematode communities using PERMANOVA (nPerm = 9999) like in [25]. We identified significant differences in community structure and then performed post-hoc tests to determine the specific environmental and biological variables driving these differences. After identifying significant environmental variables, we used db-RDA to examine relationships between soil microbial communities and other parameters (soil properties, plant and nematode communities) for each variable following the [79] methods using the R packages *ggord* V.1.0.0 [80], *pmultcomp* V. 1.4–16 [81], *factoextra* V. 1.0.7 [82] and *vegan* V. 2.5–7 [77].

#### ‘Targeted’ and ‘Global’ analysis by *Anaconda* R package for high-throughput eDNA sequencing data

The R functions created for ‘tArgeted differeNtial and globAl enriChment analysis of taxOnomic raNk by shareD Asvs’ (ANACONDA) were bottled into an R package and submitted and then published to CRAN for code review and better use by third parties [44] and can be found at https://cran.r-project.org/web/packages/Anaconda/index.html and https://github.com/PLStenger/Anaconda. This package has been created based on the data presented in this paper and was built for high-throughput eDNA sequencing analysis, but can be used for more classical ecological studies (see below with plants and nematodes data). This work package encompasses two steps: (I) the ‘targeted’ differential analysis from QIIME2 data by the DeSeq2 algorithm, and (II) the ‘global’ analysis by Taxon Mann-Whitney U test analysis from ‘targeted’ analysis. This also integrates the FunGuild [43] and Bactotraits [73] databases (for using FunGuild, Python V. > 2.7 is required).

For the first step (I), the *Anaconda* R package estimates variance-mean dependence in count/abundance ASVs data from high-throughput sequencing assays and test for differential represented ASVs (through the comparison of previously explained conditions (here in our case, F *vs*. LF, F *vs*. SF, and SF *vs*. LF) based on a model using the negative binomial distribution as in [31] for transcriptomic data (but instead of having gene expressions, we have an abundance of species). This step, therefore, focuses on whether there is an over-representation or an under-representation of specific species in one condition compared to another in a significant way. Here is a simplification of the protocol: download the R package on CRAN (https://cran.r-project.org/web/packages/Anaconda/index.html) or in its GitHub mirror (https://github.com/PLStenger/Anaconda). *i*) Use the QIIME2 files ‘*ASV*.*tsv*’ which is the list of ASVs abundance for each of your samples created by the QIIME2 pipeline; *ii*) ‘*taxonomy*.*tsv*’ which is the file with the listed taxonomy-ASV key for the rarefied dataset created by the QIIME2 pipeline (will be useful for ‘global’ analysis (II)); *iii*) ‘*taxonomy_RepSeq*.*tsv*’ which is similar to the previous file, but from the representative sequences QIIME2 step (will be useful for ‘global’ analysis (II)), and finally a handmade file named *iv*) ‘*SampleSheet_comparison*.*txt*’. More detailed material and methods can be found at https://github.com/PLStenger/Anaconda and S7 Fig. On R, the dASVa object (differential ASV abundance object) will be created to be fit on a Gamma-Poisson Generalised Linear Model (dispersion estimates for Negative Binomial distributed data), and the dispersion plot and the sparsity plot can be checked. The corresponding taxonomy can be added in the ASVs keys in results and put in a text and Excel file in output. FunGuilds can be added for fungi and Bactotrait for bacteria. MA plots are disponible in the package to adapt the *p*-value and the FoldChange cut-off.

For the second step, the ‘global’ analysis (II) by Taxon Mann-Whitney U test analysis will use the results of the ‘targeted’ analysis. This step does not specifically focus on species that are over- or under-represented in a given condition (like step I) but on all taxonomic ranks (e.g., Phylum, Class, Order, Family, Genus and Species). For this second step, more files are needed and can be downloaded here https://github.com/PLStenger/Anaconda. The first of these files, the ‘*ncbitaxon_ontology*.*obo*’, is an NCBI organismal classification file adapted for the *Anaconda* R package, originally based on [47]. The other files are a correspondence for fungi and bacteria QIIME2 code to NCBI Taxon code. Here, the Mann-Whitney U (MWU) test analysis is realised on the correspondence of the NCBI Taxon among the analogous database (NCBITaxon_MWU). This NCBITaxon_MWU uses a continuous measure of significance (such as fold-change or -log(*p*-value)) to identify NCBITaxon that are significantly enriched with either up- or down-represented ASVs. If the measure is binary (0 or 1) the script will perform a typical ’NCBITaxon enrichment’ analysis based on Fisher’s exact test: it will show NCBITaxon over-represented among the ASVs that have 1 as their measure. On the plot, different fonts are used to indicate significance, and colour indicates enrichment with either up (red) or down (blue) regulated ASVs. The tree on the plot is a hierarchical clustering of NCBITaxon based on shared ASVs. As in [41], categories that do not have any branch length separating them are included within one another. Also as in [41], the fraction next to the category name indicates the fraction of ’good’ ASVs in it; ’good’ ASVs are the ones exceeding the arbitrary absValue cutoff (option in taxon_mwuPlot()). For realised a Fisher’s based test, specify absValue = 0.5. This value does not affect statistics and is used for plotting only. The original idea was for gene differential expression analysis from [41] adapted here for taxonomic analysis (except that instead of having different functional categories of genes, we have different taxonomic ranks). This step is relevant if there is a consequent amount of data, and to hook a group of species that are taxonomically similar and present in a significant quantity in a condition.

#### *Anaconda* R package for classical ecological data

We applied *Anaconda* analyses to non-sequencing data (plants and nematodes) from classical inventories, using the ’targeted’ analysis to examine abundance files formatted to match QIIME2 *ASV*.*tsv* files (data on plants did not constitute an exhaustive database and data on nematodes stopped at family rank for the ‘global’ analysis).

## Results

### Soil eDNA pre-processing analysis

For the ITS2 marker (fungi) 2,594,514 raw sequences from 15 samples were obtained and then 270,160 sequences were kept after different cleaning steps (S1 and S2 Tables). Due to a calculated rarefaction of 12,582 reads, four plots were not kept for further analysis (namely, plots F2, LF2, LF5 and SF3) (S2 Fig). For the V4 marker (bacteria), 3,064,846 raw sequences from 15 samples were obtained and then 236,235 sequences were kept after the cleaning steps (S3 and S4 Tables). As a result of a calculated rarefaction of 4,483 reads, two samples were removed for subsequent analyses (i.e., F2 and LF2) (S3 Fig). Thus, 270,160 quality-filtered fungal sequences (ITS2) and 102,277 quality-filtered bacterial sequences (V4) from 11 and 13 soil samples respectively were finally generated and further analysed.

### Soil fungal and bacterial diversity

In total, 383 and 94 fungal and bacterial ASVs, respectively, were delineated. For both fungi and bacteria, no significant differences were observed in diversity indices between the conditions (i.e., SF, LF, and F) (S4 and S5 Figs).

### Soil fungal and bacterial composition and functional groups

**Fig 2:**
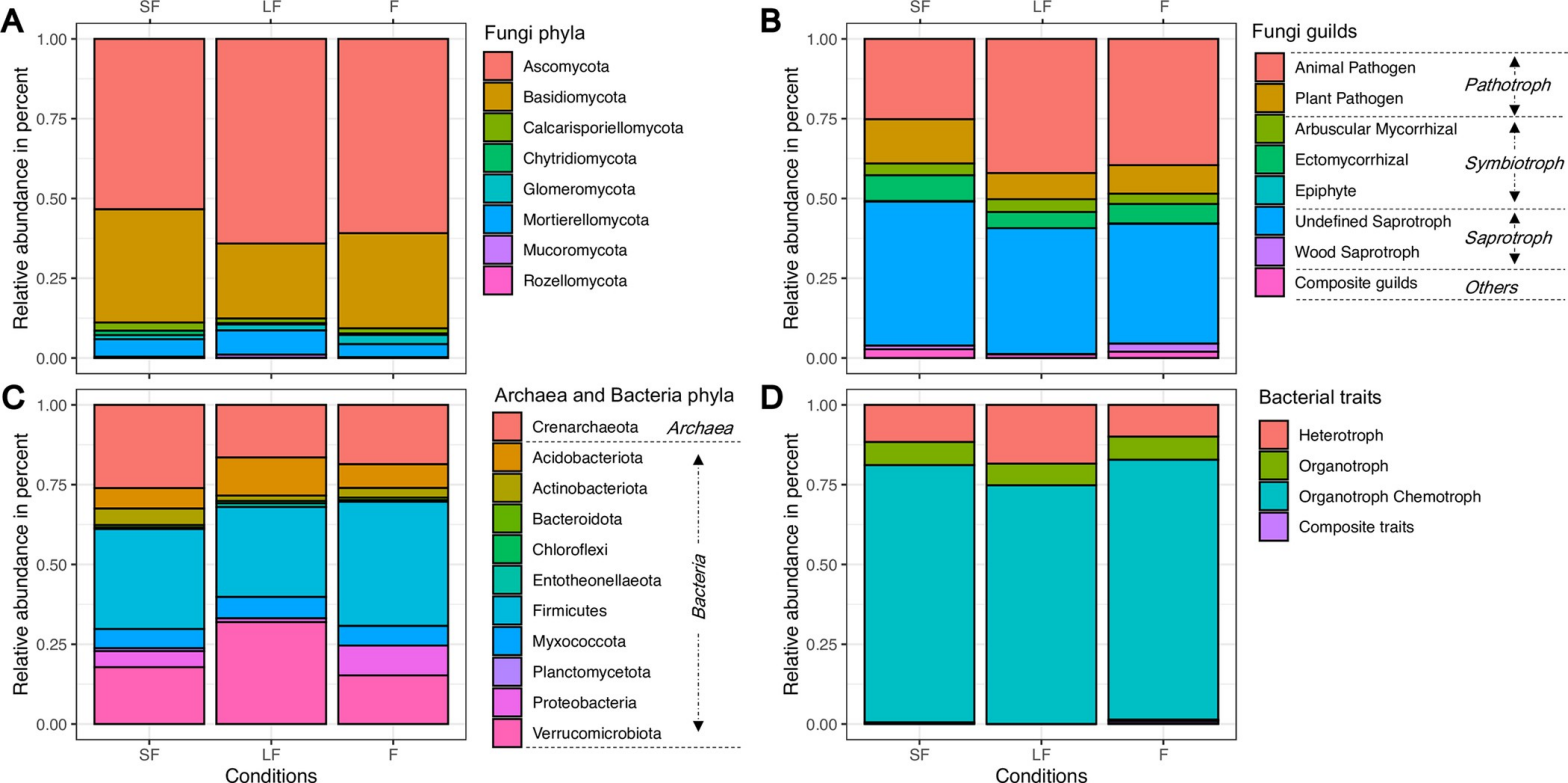
Bar plots of relative abundance (in per cent) of fungi phyla (A), their corresponding guilds (B), and of bacteria phyla (C) and their corresponding traits (D). Conditions legend: SF is for Short Fallow; LF is for Long Fallow and F is for Forest.

Fig 2 presents the relative abundances of the fungal phyla (Fig 2A) and functional groups (i.e., guilds and trophic modes) (Fig 2B). Ascomycota was observed as the most abundant phylum in each condition (SF: 55.4% ±18.6%, LF: 63.9% ±6.9%, and F: 61.5% ±8.1%), followed by Basidiomycota (SF: 33.5% ±18.5, LF: 23.9% ±7.8%, and F: 28.9% ±11.1%). All other phyla (Rozellomycota, Chytridiomycota, Mucoromycota, Calcarisporiellomycota, Glomeromycota, and Mortierellomycota) showed a relative abundance inferior to 8%. No significant variations in the proportions of the relative phyla abundances between the three conditions were detected (Kruskal Wallis test).

Regarding fungal guilds, the undefined saprotroph guild was the most relatively abundant in short fallow (44.4% ±11.3%) and long fallow (40.4% ±9.4%), and the second most abundant in the forest (36.2% ±19.1%). In the forest, the animal pathogen guild was the most relatively abundant guild with a proportion a relative abundance of 41.0% ±20.2%, whereas it was the second most abundant guild in short fallow (25.4% ± 13.8%) and long fallow, as well (40.0% ± 21.5%). The plant-pathogen guild was the third most relatively abundant guild for all conditions (SF: 13.6% ±8.3%, LF: 9.0% ±8.9%, F: 9.4% ±6.3%). The guild of ectomycorrhizal fungi was the fourth most relatively abundant guild in all conditions (SF: 8.9% ± 5.3%, LF: 5.3% ± 3.1%, F: 5.9% ± 3.6%). All other guilds showed a relative abundance inferior to 8%. When comparing the different conditions, the Kruskal-Wallis test revealed no significant variation in the relative abundances of these guilds.

The relative abundance of each bacterial phyla (and the only archaeal phyla), and their corresponding functional traits (excluding Archaea) are presented respectively in Fig 2C and 2D. For the phylum composition, in the three conditions studied, two bacterial phyla dominated the soil communities, namely the Firmicutes (SF: 31.5% ±9.3%; LF: 27.3% ±1.5%; F: 38.9% ±5.4%) and the Verrucomicrobiota (SF: 17.4% ±9.3%; LF: 32.7% ±13.3%; F: 15.3% ±%5.4). The only detected archaeal phylum that was the Crenarchaeota, was also observed in relatively high proportions (SF: 25.9% ±12.5%; LF: 16.8% ±8.4%; F: 18.6% ±2.5%) (Fig 2C). All these phyla did not show any significative differences in their relative abundances between the three compared treatments. The only phylum that presented significant variations in its proportions (SF: 4.9% ±0.6%; LF: 1.2% ±1.4%; F: 9.4% ±2.3%) was the Proteobacteria (Kruskal Wallis test *p-*value = 0.004723).

Concerning the bacterial functional traits, the organotroph-chemotroph functional group was dominant in all conditions (SF: 80.4% ±2.7%, LF: 72.0% ±11.2%, and F: 80.9% ±2.1%), and was followed by the heterotroph group (SF: 11.7% ±2.7%, LF: 21.5% ±12.2%, F: 10.4% ±2.5%). The organotrophs and the composite group represented systematically less than 8%. The heterotrophs were the only functional group that showed a significant variation between conditions (Kruskal Wallis test, *p-*value = 0.0333).

### Microbial communities’ structure

**Fig 3:**
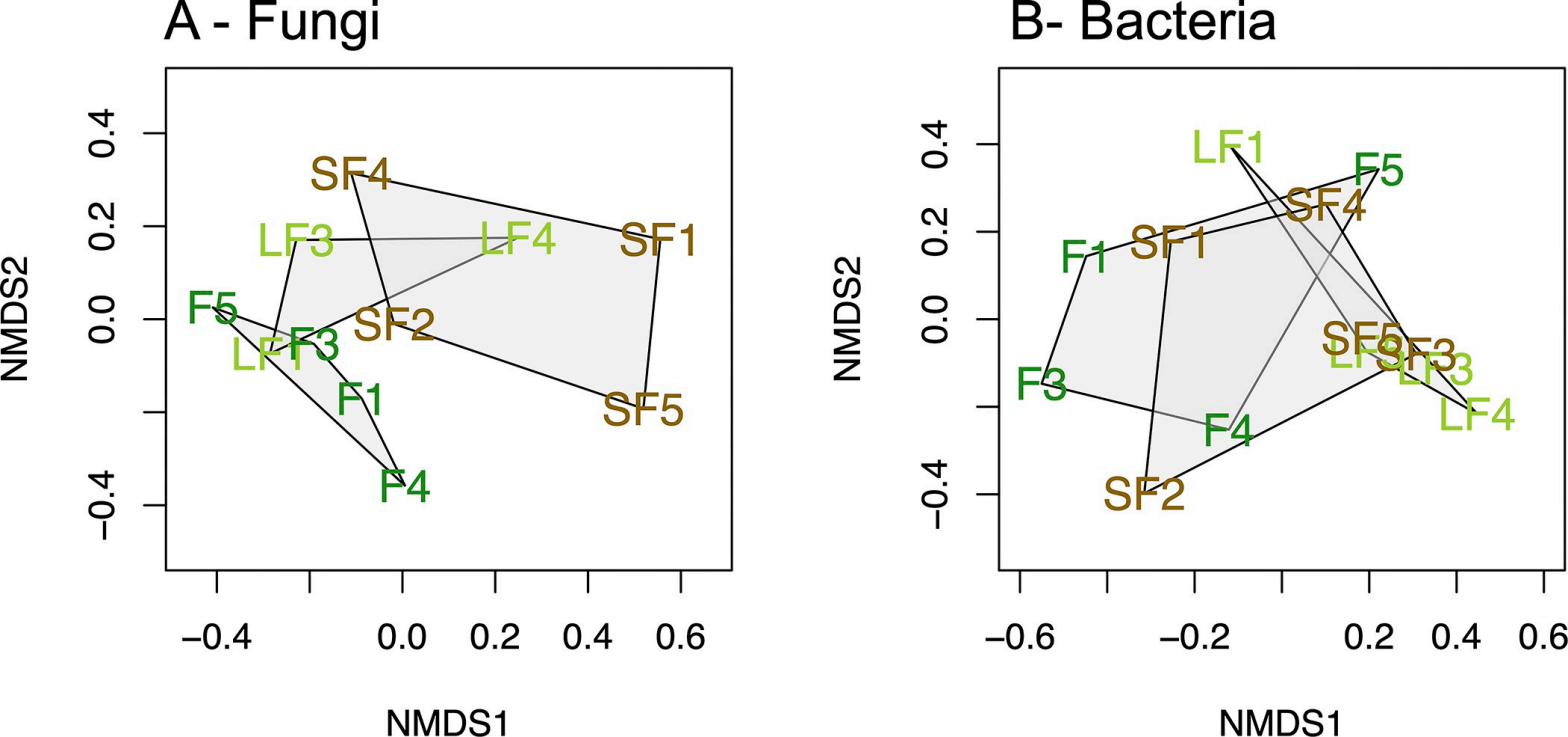
Non-metric multidimensional scaling (NMDS) for fungi phyla (A) and bacteria phyla (B). SF is for Short Fallow (brown letters); LF is for Long Fallow (light green letters) and F is for Forest (forest green letters).

The NMDS ordination, based on the Bray-Curtis dissimilarity index, suggests that soil fungal communities were distinct between the studied conditions, particularly between short fallow and forest (Fig 3A). The PERMANOVA analysis supports this observation (PERMANOVA: *p-*value = 0.018, R^2^ = 0.277 for sites and 0.723 for residual, post-hoc test pairwise adonis F *vs*. SF *p-*value = 0.019; F *vs*. LF and LF *vs*. SF were non-significant). Conversely, for bacteria, no community structure was observed (Fig 3B; PERMANOVA non-significant). Thus, in contrast to fungi, bacterial communities did not exhibit any significant differences between land-use conditions.

### Influence of physico-chemical parameters

The soil texture (*i*.*e*., the proportions of clays, silt, and sand) was homogeneous among plots and, hence, among the three related conditions investigated (S5 Table, S6 and S8 Figs) and was classified as silt loam. The organic matter, carbon (C), nitrogen (N), and pH showed significant differences (*p-*values < 0.05), with systematically higher values in the forest (S5 Table and S8 Fig). In linked organic matter analysis, C/N showed significant differences (*p-*value = 0.029), with a higher ratio in long fallow. Regarding the microbial biomass parameters, significant differences were observed (S5 Table and S8 Fig). Indeed, the carbon, the estimated total microbial biomass, as well as the estimated related parameters nitrogen, phosphorus, potassium, calcium, and magnesium stored in microbial biomass showed significant differences between conditions (*p-*values < 0.05). Looking at the pairwise comparisons, the soils in forests presented, for most parameters, higher significant values. In addition to these investigated parameters, significant differences were found in mineralized carbon (microbial activity), decreasing from forest to short fallow (*p*-value = 0.015, S5 Table; S8 Fig). Since a structure of communities according to the studied conditions was only observed for fungi (Fig 3), db-RDA analysis using soil physico-chemicals parameters as explanatory variables has only been proceeded on this microbial group. The db-RDA representation showed that fungal phyla were significantly related to the soil texture and not to other parameters (S9 Fig–with only the significant parameter, i.e., the soil texture). The PERMANOVA (nPerm = 9999; 20% of the variance explained, *p-*value = 0.007) and post-hoc tests (Clays, *p-*value = 0.031; and Silt, *p-*value = 0.009) supported this relationship. More precisely, the Basidiomycota were found to be related to the silt content, whereas the Ascomycota were inversely related to the clay content (S9 Fig). However, no relationships were detected regarding the soil fungal fallows and forest communities. So, the soil texture was homogeneous among plots, but the soil physico-chemical properties, including organic matter, carbon, nitrogen, and pH, showed significant differences between the forest and fallow conditions, with the forest having systematically higher values.

### Influence of plants

Kruskal-Wallis tests, followed by Dunn post-hoc tests (S6 Table) revealed significant differences in plant species composition between the forest and fallow conditions, with several species showing significant presence or absence in specific conditions, such as *Acacia spirorbis* (*p-*value = 0.00621) being absent in the forest and *Dodonaea viscosa* (*p-*value = 0.00327) being more present in short fallow.

### Influence of nematodes

One nematode family, Aphelenchoididae (Kruskal-Wallis test: p-value = 0.00918), showed significant changes between conditions, with differing abundance in long-term and short-term fallows (S7 Table).

### ‘Targeted’ analysis for fungi and bacteria with the *Anaconda* R package

**Fig 4:**
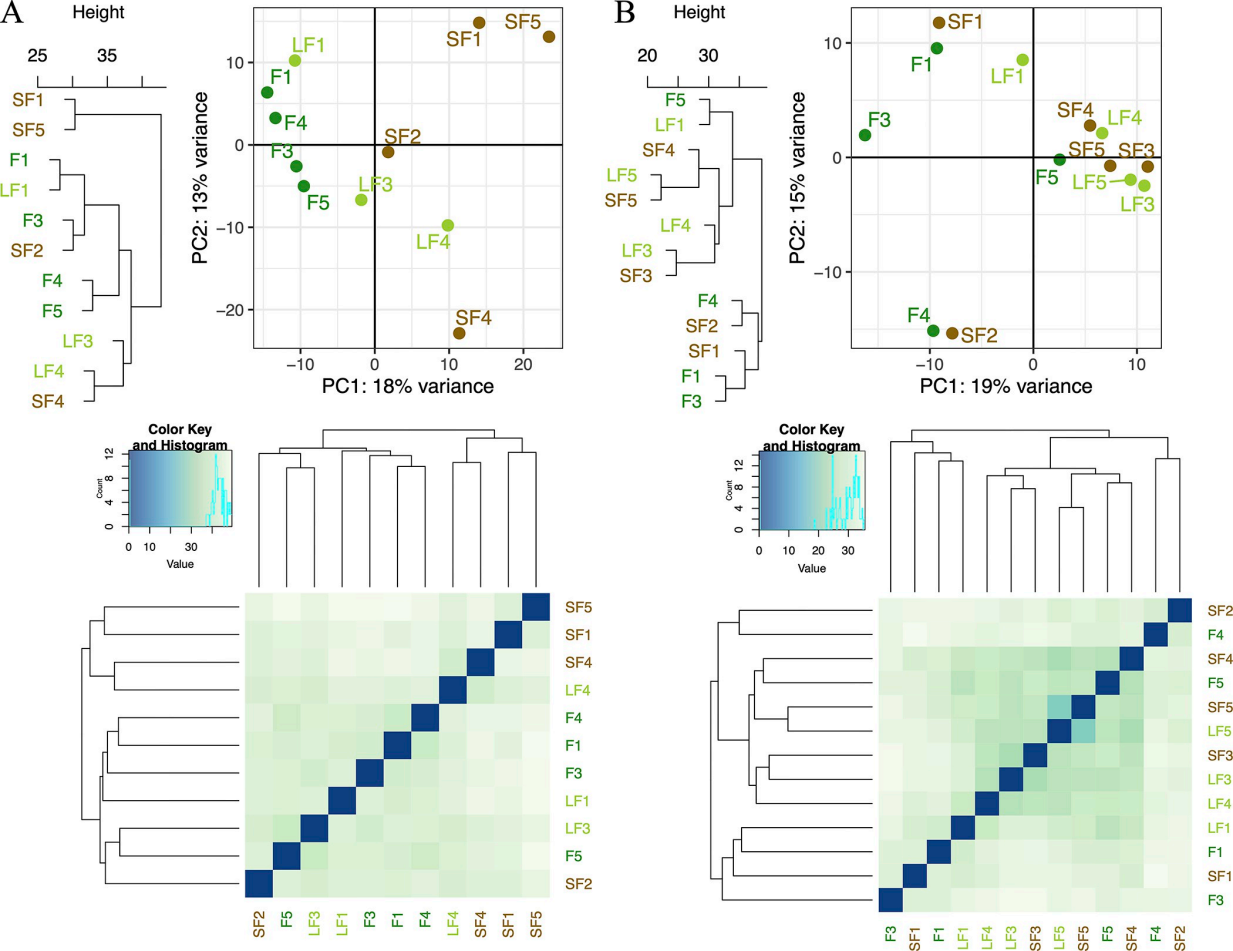
Analysis of the inter-sample relationships for fungi (A) and bacteria (B) with the *Anaconda* R package. For each, at the left, a hierarchical clustering on Euclidean distances on logarithm-transformed ASVs abundance with average clustering method, at the right a Principal component analysis (PCA), and at the bottom, a sample-to-sample heatmap based on rlog transformation with trim on too low represented ASVs. SF is for Short Fallow (brown letters); LF is for Long Fallow (light green letters) and F is for Forest (forest green letters).

Eleven and 13 samples were used, respectively, for fungi and bacteria/archaea analyses (as a result of the deletion of samples due to the previous rarefaction step). An estimate of the dispersion by shrinkage can be visualised by plotting the dispersion estimates on the average ASVs presence strength (here ‘ASV abundance’ is used as a ‘count’) (S10 Fig) by adjusting only an intercept term. First, and following [31], the maximum likelihood estimate of the ASVs was obtained using only the respective ASVs data (black dots). Then, a curve (red) was fitted to the maximum likelihood estimate to capture the general trend of the dispersion-mean dependence. This fit was used as a prior mean for a second round of estimation, which resulted in the final estimates of the dispersion at the maximum a posteriori. This can be understood as a narrowing (blue circle) of the noisy estimates by ASVs towards the consensus represented by the red line. The black points circled in blue were detected as outliers of the dispersion and were not reconciled with the prior (the reconciliation would follow the dotted line). In our case, we see that few ASVs were not fitted in the (here, parametric) model (which is normal according to [31]) and that the results were very similar between the two kingdoms, although the bacteria showed few ASVs in comparison. The analysis of the inter-sample relationships after the previous transformation (Fig 4) showed us that the variability observed in the previous analyses (e.g., sections 3.2 to 3.4) was well preserved. For example, the similarity between the NMDS (Fig 3) and the PCA presented here was remarkable. Nevertheless, we can observe nuances in this variability.

For fungi (Fig 4A), the hierarchical clustering on Euclidean distances on logarithm-transformed ASVs abundance with average clustering method showed higher differences in sample relationships than in the PCA. As an example, the samples from the Forest condition (‘F’) were tightly grouped in this PCA whereas they were fitted in three different sub-clusters in the hierarchical clustering. The 31% (18%+13%) total explained variation in the PCA showed that a small part of the data explained this convergence. The sample-to-sample heatmap based on rlog transformation with trim on too low represented ASVs showed a certain homogeneity of the samples, which could illustrate a variability homogeneously explained by some ASVs (with a variability not pulled by only some ASVs in a specific way, but also by several ASVs in the same direction).

For bacteria and archaea (Fig 4B), the hierarchical clustering on Euclidean distances on logarithm-transformed ASVs abundance with average clustering method showed similar differences in sample relationships than in the PCA. As an example, the samples F1, F3, F4, SF1, and SF2 were at the margin of the other samples in the PCA, which was well highlighted by a similar sub-cluster in the hierarchical clustering. The 34% (19%+15%) total explained variation in the PCA showed that a small part of the data explained the presented variation. The sample-to-sample heatmap based on rlog transformation with trims on too low represented ASVs, showed higher heterogeneity within some of the samples, as for LF5-SF5 for example, this could display a variability explained by few ASVs in a heterogeny way (with a statistical variation pulled by few ASVs, and not a lot of ASVs). Such similarities (e.g., between the previous and the *Anaconda* analyses) with nuances allowed us to ensure that the variability structure of the dataset (e.g., few ASVs highly over or under-represented in a condition *versus* a lot of similar ASVs slightly over or under-represented in a condition) was maintained while allowing us to explore the smallest variation so that we can answer our biological/ecological question with further analysis (see below).

The clustered heatmap of the 75 most abundant ASVs based on Euclidean distance with average clustering method for fungi (S11 Fig) and bacteria/archaea (S12 Fig) showed that there is no discernible pattern based on the most prevalent ASVs. This allows for condition-specific analyses to recover ASVs that are specifically over- or under-represented in the different conditions, which could therefore explain the observed variations.

**Fig 5:**
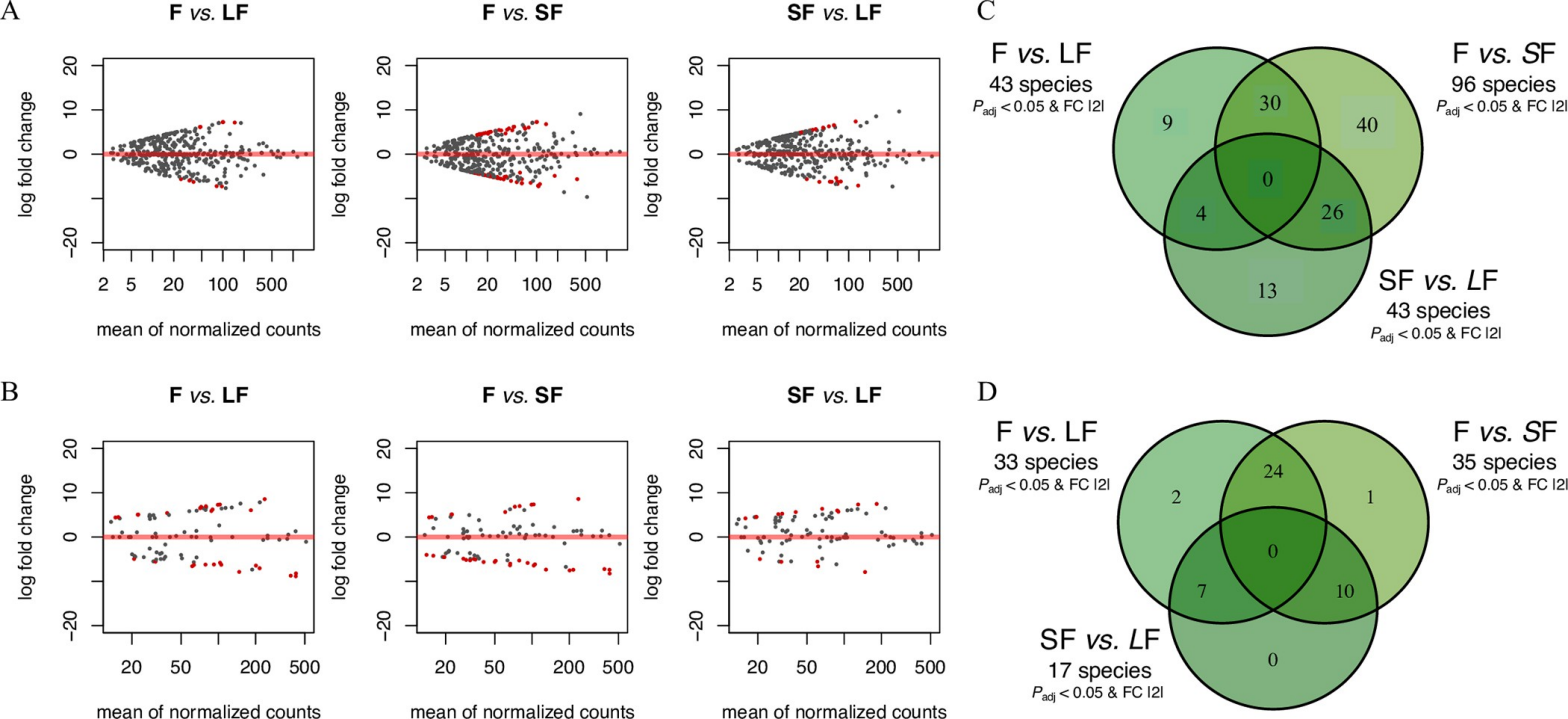
MA plots (A and B) from the mean of normalised count by their respective log fold change, and Venn diagrams from ASVs (C and D) realised for fungi (A and C) and bacteria (B and D) with the *Anaconda* R package. SF is for Short Fallow; LF is for Long Fallow and F is for Forest.

The DeSeq2 algorithm allowed such comparison, and here with *P-*adjusted < 0.05 and LogFoldChange > |2|, F *vs*. LF, F *vs*. SF, and SF *vs*. LF comparison hooked, respectively, 43, 96, and 43 significantly under- or over-represented ASVs respectively for fungi (Fig 5A), and 33, 35, and 17 significantly under- or over-represented ASVs for bacteria (Fig 5B).

Venn diagram representations (Fig 5C and 5D) allowed us to recover species (ASVs) that were *i*) specific to a comparison, and most importantly that were *ii*) specific to a condition (the latter correspond to those with a common denominator, e.g., F *vs*. SF compare to F *vs*. LF will show ASVs specific to F). For fungi (Fig 5C), of the 43 ASVs significantly over- or under-represented in the F *vs*. LF pairwise comparison, nine were specific to this comparison. Out of the 96 ASVs significantly over- or under-represented in the F *vs*. SF comparison, 40 were here specific to this comparison. For the SF *vs*. LF comparison, of the 43 ASVs significantly over- or under-represented, 13 were specific to this pairwise comparison. Thirty ASVs, 4 ASVs, and 26 ASVs significantly over- or under-represented were specific to the forest, the long fallow and the short fallow, respectively (condition-specific ASVs). For the bacteria/archaea (Fig 5D), of the 33 ASVs significantly over- or under-represented in the F *vs*. LF comparison, two were only recovered from this comparison. Out of the 35 ASVs significantly over- or under-represented in the F *vs*. SF comparison, only one was specific to this pairwise comparison. Of the 17 ASVs significantly over- or under-represented in the SF *vs*. LF comparison, none were specific. Twenty-four, 7, and 10 ASVs that were significantly over- or under-represented were, respectively, restricted to the forest, the long fallow and the short fallow (condition-specific ASVs).

**Fig 6:**
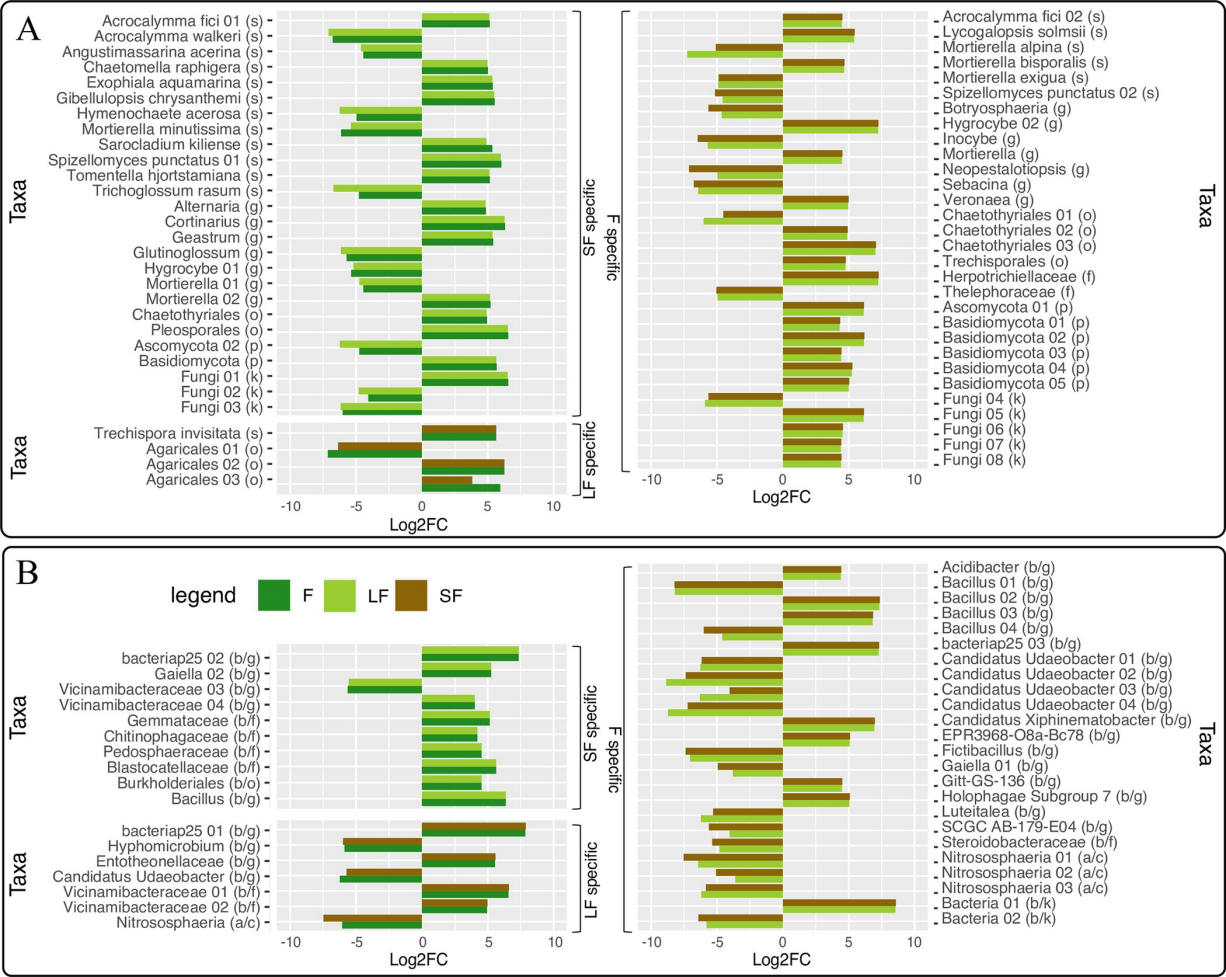
‘Targeted’ analysis results graph for fungi (A) and bacteria (‘b’) and Archaea (‘a’) (B) with the *Anaconda* R package. Results are in Log2FC for the significant ASVs according to the precision of the taxonomic rank: species (‘s’), genus (‘g’), family (‘f’), order (‘o’), class (‘c’), phyla (‘p’), or kingdom (‘k’). SF is for Short Fallow (brown); LF is for Long Fallow (lightgreen) and F is for Forest (forestgreen).

Looking at the fungal ASVs (*p-*value < 0.05; LogFoldChange > |2|) that were condition-specific (Fig 6A), 15 ASVs were over-represented in short fallows (present only in short fallows), particularly *Sarocladium kiliense*, *Acrocalymma fici* and *Exophiala aquamarina*, and 11 were under-represented in short fallows (present in forests and long fallows, but not in short fallows), notably *Acrocalymma walkeri*, *Angustimassarina acerina*, and *Mortierella minutissima*. In long fallows condition-specific, three ASVs were found to be over-represented, such as *Trechispora invisitata*, and one was under-represented, namely *Agaricales sp*. *01*. In forests condition-specific, 20 ASVs were over-represented, like *Mortierella bisporalis*, *Lycogalopsis solmsii* and *Hygrocybe sp*.), and 10 were under-represented, like *Spizellomyces punctatus*, *Botryosphaeria sp*. and *Mortierella alpina*.

For ASVs (*p-*value < 0.05; LogFoldChange > |2|) that were condition-specific for bacteria and archaea (Fig 6B), nine ASVs were over-represented, such as Burkholderiales, Gemmataceae, or Gaiella, and one was under-represented in short fallows, an ASV assigned at the Vicinamibacteraceae family. In long fallows, four ASVs were over-represented, for instance, bacteriap25 sp., Entotheonellaceae sp. and Vicinamibacteraceae sp., and three were under-represented, *i*.*e*., Hyphomicrobium sp., *Candidatus Udaeobacter* and Nitrososphaeria sp. (archaea). Finally, nine ASVs were over-represented, like *Candidatus xiphinematobacter*, *Bacillus sp*. and *Acidibacter sp*., and 15 were under-represented in F, such as three archaeal ASVs belonging to the Nitrososphaeria genus.

So here, hierarchical clustering analysis revealed greater differences in fungal sample relationships, with distinct sub-clusters in Forest samples, while PCA showed a more compact grouping. Bacterial and archaeal samples exhibited similar patterns, with some distinct sub-clusters and others showing higher heterogeneity. Condition-specific ASVs were identified in fungi and bacteria/archaea, with distinct ASVs over- or under-represented in each condition, including *Sarocladium kiliense*, *Acrocalymma fici*, and *Exophiala aquamarina* in short fallows, and *Mortierella bisporalis*, *Lycogalopsis solmsii*, and *Hygrocybe* sp. in forests.

### ‘Global’ analysis of fungal and bacterial communities with the *Anaconda* R package

**Fig 7:**
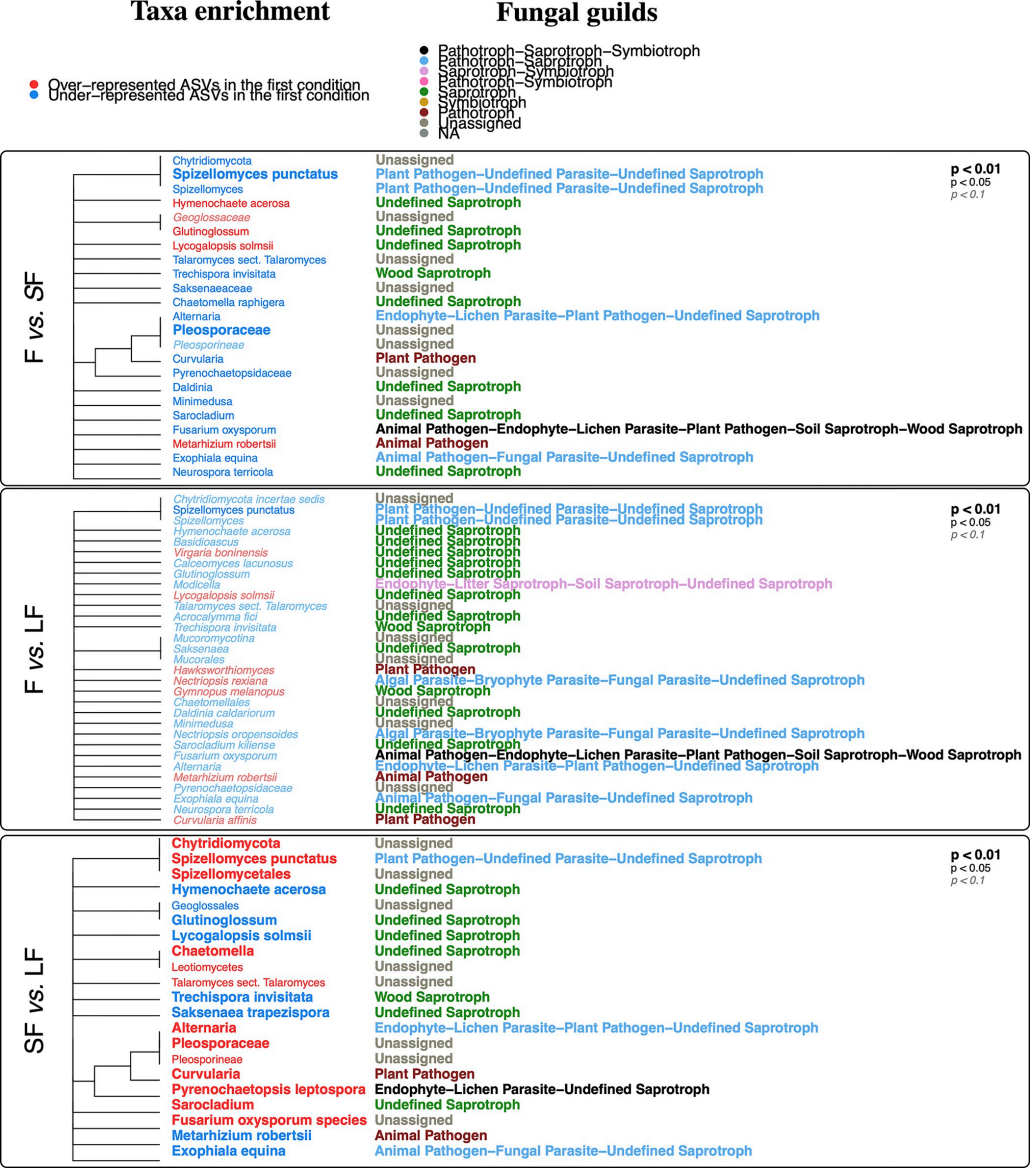
‘Global’ analysis results graph for fungi with the *Anaconda* R package for the three comparisons. SF is for Short Fallow; LF is for Long Fallow, and F is for Forest.

Concerning the ‘global’ analysis for fungi (Fig 7), 1174 ASVs matched to 653 NCBITaxon different terms, and 639 NCBITaxon were remaining. After the secondary clustering, the MWU test output 23, 31, and 21 NCBITaxon terms at 10% FDR for the pairwise comparisons F *vs*. SF, F *vs*. LF, and SF *vs*. LF, respectively. Here, compared to the ‘targeted’ analysis, when an affiliation is made at a higher rank than species (e.g., family, order, or genus), this corresponds to several ASVs that share the same taxonomic rank. When a group of ASVs are ascribed at the species level, it means that several ASVs share this taxonomic affiliation and can correspond to different sub-species, or strains.

In the forest, compared to the short fallow, numerous ASVs assigned to the fungal entomopathogen *Metarhizium robertsii* were over-represented (*p-*value < 0.05) (Fig 7). In contrast, other ASVs were under-represented, hence, over-represented in the short fallow, such as the ones ascribed to the *Curvalaria* plant pathogen genus (*p*-value < 0.05), the potential plant pathogens that are *Spizellomyces punctatus* (*p-*value < 0.01) and *Fusarium oxysporum* (*p-*value < 0.05), and the potential human pathogen that is *Exophiala equina* (*p-*value < 0.05) (Fig 7).

In comparison to the long fallow, *Metarhizium robertsii* was again found in higher abundance than in the forest. Only *Spizellomyces punctatus* was significantly observed as over-represented (*p-*value < 0.05) in the long fallow, but to a lesser extent than in the short fallow (Fig 7).

The comparison between the short and the long fallow periods (Fig 7) showed an over-representation in the former for numerous taxa: *Sarocladium*, *Fusarium oxysporum* species complex, *Pyrenochaetopsis leptospora*, *Curvularia*, Pleosporaceae, *Alternaria*, *Chaetomella*, *Chytridiomycota*, and *Spizellomyces punctatus* (*p-*value < 0.01). *Leotiomycetes* and *Talaromyces* sect. *Talaromyces* were also over-represented in the short fallow (*p-*value < 0.05). In the long fallow, *Hymenochaete acerosa*, *Glutinoglossum*, *Lycogalopsis solmsii*, *Trechispora invisitata*, *Saksenaea trapezispora*, *Metarhizium robertsii*, and *Exophiala equina* were found in higher abundances than in the short fallow (*p-*value < 0.01).

For bacteria and archaea, 486 ASVs matched to 108 NCBITaxon different terms. One hundred NCBITaxon were remaining. After the secondary clustering, the MWU test output zero NCBITaxon terms at 10% FDR for all comparisons. This result mirrors the ones presented in Fig 4, which displays a variability explained by a few ASVs in a heterogeny way (with a variability pulled by only some ASVs in a specific way, and not by several ASVs in the same direction). Here it means that some ASVs are very strongly over- or underrepresented in a condition (which is why the ‘targeted’ analyses worked) but that there are not enough similar ASVs that are slightly over- or underrepresented in a condition in the same direction (which is why the ‘global’ analyses cannot be realised because it is not significant).

To summarize, the analysis of fungal ASVs in the forest, short fallow, and long fallow conditions revealed significant differences in taxonomic affiliations, with *Metarhizium robertsii* being over-represented in the forest and short fallow, and *Curvalaria*, *Spizellomyces punctatus*, and *Fusarium oxysporum* being under-represented in the forest and over-represented in the short fallow, whereas *Leotiomycetes* and *Talaromyces* sect. *Talaromyces* were over-represented in the short fallow. The analysis of bacterial and archaeal ASVs revealed that only 108 taxonomic groups were significantly represented, with the majority of ASVs remaining unclassified, and no significant differences were found between the forest, short fallow, and long fallow conditions, indicating that a few ASVs strongly drive the variation in this community composition.

### *Anaconda* package on ecological data

**Fig 8:**
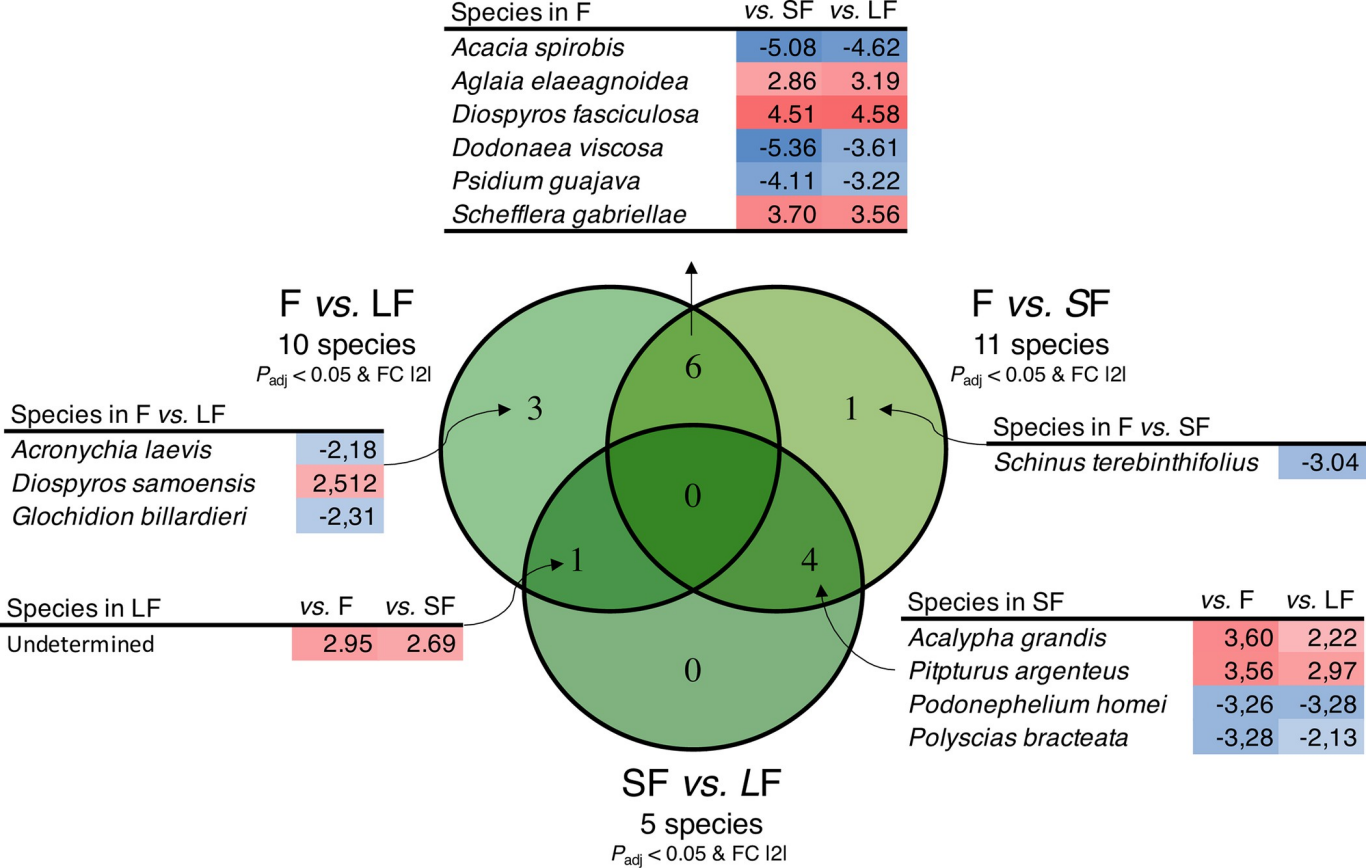
Venn diagram from the ‘targeted’ analysis results graph for plantae for the three comparisons. Values are corresponding fold changes. Conditional blue values are under-represented ASVs (negative fold change) and conditional red values are over-represented ASVs (positive fold change). SF is for Short Fallow; LF is for Long Fallow, and F is for Forest.

To determine the usefulness of this tool for other types of data, the *Anaconda* package was used on plants’ and nematodes’ ecological data. Using the ‘targeted’ analysis, six plant species were observed as forest-specific (*p-*value < 0.05; LogFoldChange > |2|), notably *Aglaia elaeagnoidea*, *Diospyros fasciculosa*, and *Schefflera gabriellae* (Fig 8). *Acacia spirorbis*, *Dodonaea viscosa*, and *Psidium guajava* were only encountered in the two fallowing periods (Fig 8). *Acalypha grandis* and *Pitpturus argenteus* were only observed in the short fallow (*p-*value < 0.05; LogFoldChange > |2|), whereas *Podonephelium homei* and *Polyscias bracteata* were only found in the two other conditions. *Schinus terebinthifolius* was found significant in the short fallows compared to the forests (*p-*value < 0.01), which means that this species was found in larger quantities in short fallows compared to forests. In forests *vs*. long fallows, *Acronychia laevis* (*p-*value = < 0.01) and *Glochidion billardieri* (*p-*value = < 0.05) were encountered in greater quantities in long fallows compared to forests, meaning that these species were mostly found in long fallows compared to forests. Finally, *Diospyros samoensis* (*p-*value = < 0.05) was observed in higher abundance in forests compared to long fallows, indicating that it was mostly present in the forest and slightly in the long fallow land.

For nematodes, the comparison between the forest and the short fallow showed significant variation in the abundance of one family, the Tylenchidae, which was more present in the forest (*p-*value = 0.01665, LogFoldChange = 2.28). A significant variation in another family, the Aphelenchoididae, was also observed when the short and low fallow were compared, with a higher representation in the former (*p-*value = 0.00974, LogFoldChange = -2.23).

So here, the application of the ‘targeted’ analysis to plant and nematode ecological data revealed significant differences in species abundance between the forest and fallow conditions, with specific plant species showing preferences for either forest or fallow environments, and nematode families exhibiting altered abundance patterns in response to different land-use regimes. This clearly demonstrates the usefulness of this package for ‘classic’ ecological data, and its use can therefore be extended beyond metabarcoding data.

## Discussion

### ‘Classical’ community analysis: No effect of agricultural practice changes in the first instance

The so-called ‘classical’ community analysis (which refers to the diversity, composition, and structure investigations that are commonly made in community analyses), revealed no effects of cultural practice changes on soil microbial communities. Indeed, no differences in microbial diversity were found between short- and long-term fallowing, and forest, for both fungi and bacteria/archaea. Variations in phylum composition were only observed for Proteobacteria, with a higher proportion in the forest, but not between fallow periods. Based on [25], the relative abundance of Proteobacteria may indicate soil and land degradation, suggesting that both short- and long-fallow periods (the latter lasting over a decade) could be considered as degraded systems. As for the diversity and the phyla composition, the functional groups did not reveal a clear tendency, especially in terms of pathogens and beneficial microbe relative abundances. Looking at the soil microbial community structure, a significant partitioning was only observed for fungi, which resulted from differences with the forest, not from any fallowing period effect.

It’s noteworthy that despite our inability to detect soil microbial changes due to the agricultural practice, some ‘global’ tendencies seem to emerge from those ‘classical’ approaches. Indeed, for fungi, in all conditions Ascomycota was observed as the most abundant phylum, followed by Basidiomycota. In the literature, the dominance of Ascomycota over Basidiomycota has been recently suggested as an indicator of ecosystem degradation [25]. This may thus suggest that even the forests used as reference ecosystems are in some extent degraded. Regarding bacteria, the Firmicutes and Verrucomicrobiota phyla dominated the soil communities. The Firmicutes have been classified as copiotrophic [83] and Verrucomicrobiota as oligotrophs [84]. However, a recent study has conversely shown a positive correlation between Verrucomicrobiota and soil carbon content [85]. The high soil organic carbon content of Maré’s Gibbsic Ferrasols, even in cultivated soils [49], could thus be a probable explanation for the over-representation of these two bacterial groups. In addition to these bacterial phyla, the Crenarchaeota was also well-represented in all conditions and was the only archaeal representative. The dominance of archaeal communities by Crenarchaeota on Maré island is in accordance with the observations made by [86] on divers’ soils at a worldwide scale. This group may play central roles in biochemical cycles in soils [86, 87]. However, deeper investigations are needed to better understand the roles of microorganisms in Gibbsic Ferralsols on Maré island. Indeed, except soil texture, environmental variables were not found to influence soil microorganisms.

At this stage, based on “classical” analyses, we cannot conclude that changing agricultural practices at Maré Island have any effect on soil microbial communities. We cannot rule out a lack of effect, but we can also acknowledge the need for more in-depth approaches to highlight potential changes, particularly in the soil health and One Health context.

### Revolutionising soil health and One Health through advanced detection of soil pathogens with the *Anaconda* package

The two newly developed statistical analyses implemented in the *Anaconda* package, namely the ‘targeted’ and ‘global’ analyses, highlighted the over-representation of microbial ASVs, particularly for fungi, ascribed to plant and animal pathogens, including humans, in the short fallow. Indeed, fungal pathogens such as *Acrocalymma fici*, known as a pathogen of cultivable trees [88], *Chaetomella raphigera*, recognised as a fruit rot pathogen [89], and *Gibellulopsis chrysanthemi*, identified as a root rot pathogen [90] were detected in significantly higher proportions through the ‘target’ approach in the short-term fallow (Fig 6). Additionally, an undetermined species belonging to the *Botryosphaeria* genus, a taxon known to be associated with grapevine decline [91], was absent in the forest and present in both fallows, with higher abundance in the short-duration fallow. In congruence with all these results, an increase of plant fungal pathogens in the short fallow was observed using the ‘global’ statistical investigation. For instance, taxa such as *Fusarium oxysporum*, *Alternaria* and *Curvularia*, known to be pathogenic to many plant species [92–94], were particularly present in the short fallow compared to both the long fallow and the forest ecosystem.

In addition to these plant-detrimental microbes, a fungal taxon of primary interest for Human health has also been detected in the short fallow soils, namely *Sarocladium kiliense* (formerly *Acremonium kiliense*). *S*. *kiliense* is a soil saprophytic fungus that can cause opportunistic infections in immunocompetent and immunocompromised individuals, with diverse manifestations, such as dermatophytosis, onychomycosis, mycetoma, pneumonia and fungemia [95, 96]. Outbreaks of *S*. *kiliense* in immunodepressed patients have been reported in the literature [97–99]. These clusters were likely linked to infections in clinical settings [98, 99], but a probable environmental source has also been suggested by [97]. Recently, a fatal disseminated infection in a diabetic patient with coronavirus disease 2019 (COVID-19) has been reported by [100] in Iran. The severity of the diseases that can result from *S*. *kiliense* underlines the necessity of a high level of clinical attention in this area. In New Caledonia, at the public hospital, hitherto two cases involving undefined *Sarocladium* species have been reported (data on geographical origin and patient health non-available) (Arnaud Cannet, pers. com.). In light of the aforementioned fatal case in Iran [100], *Sarocladium* risk infections have to be in regards to the substantial diabetic population in New Caledonia (ASSNC, 2022), as well as the high prevalence of COVID-19 in the archipelago (WHO Coronavirus (COVID-19) Dashboard, https://covid19.who.int/). The ‘global’ analysis of this study shows an over-representation of the genus in the comparison of short *versus* long fallows, which also confirms the results of the ‘targeted’ analysis. The over-representation of this harmful fungus in a traditional agricultural system could result in a higher probability of infection and, therefore, support the need to raise awareness about this pathogen among healthcare workers and the local populations.

From the ‘targeted’ analysis (Fig 6), *Exophiala aquamarina*, an opportunistic fungal pathogen causing cutaneous and disseminated infections in cold-blooded vertebrates (so far restricted to fishes) [101], was also found to be significantly over-represented in the short fallow. Based on the ‘global’ approach (Fig 7), another *Exophiala* species in the same phylogenetic clade, *E*. *equina*, was significantly present in soil samples from both short-term and long-term fallows, with greater representation in the latter. This suggests that agricultural establishment, regardless of the fallowing period, increased this pathogen in Maré’s soils. Similar to *S*. *kiliense*, this underscores the need to monitor potential human infections by *E*. *equina*, which, although rare, can cause cutaneous and subcutaneous infections [101, 102]. Supporting the necessity of paying attention to this genus, two cases of *Exophialum* infections have been to date reported for the public hospital in New Caledonia (data not available on the geographical origins of the patients) (Arnaud Cannet, pers. com.).

Regarding bacteria, the *Anaconda* results were less clear than for fungi. Despite no findings from the ‘global’ analysis, likely due to high intra-sample variability, the ‘targeted’ analysis identified ASVs in the short fallow attributed to taxonomic groups containing or suspected of containing pathogens, such as the Gemmataceae (Planctomycetes) [103] and Burkholderiales [104] (Fig 6). Indeed, molecular-based detection has revealed the presence of Planctomycetes in the blood of leukemic of two aplastic patients with neutropenia, rash, diarrhoea and micronodular pneumonia [105]. The phylogenetic analysis revealed for one of the clinical cases a close relationship to *Gemmata obscuriglobus*, a species that belongs to the Gemmataceae. For the second case, according to [106], when sequences of the 16S rRNA gene were compared, the second hit with a described taxon was with another *Gemmata* species, *G*. *massiliana*. This bacterium was originally recovered and characterised from a hospital water distribution system in France [107], thus in proximity to patients, as pointed out by [103]. *Gemmata*-related sequences have also been found in human stool specimens, including individuals with infective endocarditis [108]. From these constatations and other cellular and molecular findings, Gemmataceae representatives which clinical microbiologists have overlooked [106], have been suggested to potentially behave as opportunistic pathogens [103, 106]. Concerning the Burkholderiales (given as a second example), it encompasses a large variety of organisms, in particular plant and animal pathogens, including for humans [109]. Certain Burkholderiales bacteria are considered particularly dangerous for individuals suffering from chronic lung diseases [104].

### Uncovering beneficial soil microbes and ecological links with *Anaconda’*s statistical approaches

As just seen above, the approach implemented in the *Anaconda* package revealed an increase of soil microbial pathogens with a reduction in the fallowing period. In complement to this compelling constatation, other soil microorganism types that displayed differences in their occurrence and deserve great attention were also recovered from *Anaconda* analyses. Indeed, several fungal saprophytes, *i*.*e*., *Glutinoglossum sp*, *Hymenochaete acerosa*, *Lycogalopsis solmsii*, *Trechispora invisitata* and *Sakseneae trapezispora*, were detected in lower prevalence in the short fallow’s soils (Figs 6 and 7). It has been shown that saprophytic fungi can be involved in the regulation of pathogens [92, 110, 111]. Competition for resources [92] and antagonist interactions, *via* saprophyte fungi promoting soil antifungal bacteria [110, 111], are underlying mechanisms leading to soil pathogen suppression. The lowest value of mineralised carbon in the short fallow, which reflects a lower microbial activity, argues in favour of a diminution of saprophyte activity. We could, thereby, hypothesise that the specific decrease of these saprophytic fungi has favoured the increase of the detrimental microorganisms observed in the short fallow plots. Alongside saprophytes, the fungal animal pathogen *Metarhizium robertsii* was in a decreasing order well-represented in the forest, then in the long and the short fallow (Fig 7). This fungus is an entomopathogen infecting a wide range of arthropods, and can consequently be involved in insect pests’ regulation [112]. It can also establish itself as a root endophyte and favour plant growth and defence against plant pathogens [113]. The specific lower abundance of this entomopathogenic and plant-endophyte fungus in the short fallow may similarly favour an increase of detrimental organisms. Thus, in the context of soil suppressiveness (*i*.*e*., the capacity of any given soil to reduce pathogens and disease incidence), specific suppression mechanisms, through individual species or selected groups of antagonist microorganisms [17, 114, 115], seem to regulate soil borne-pathogens in our system, rather than microbial diversity [7, 115].

**Fig 9:**
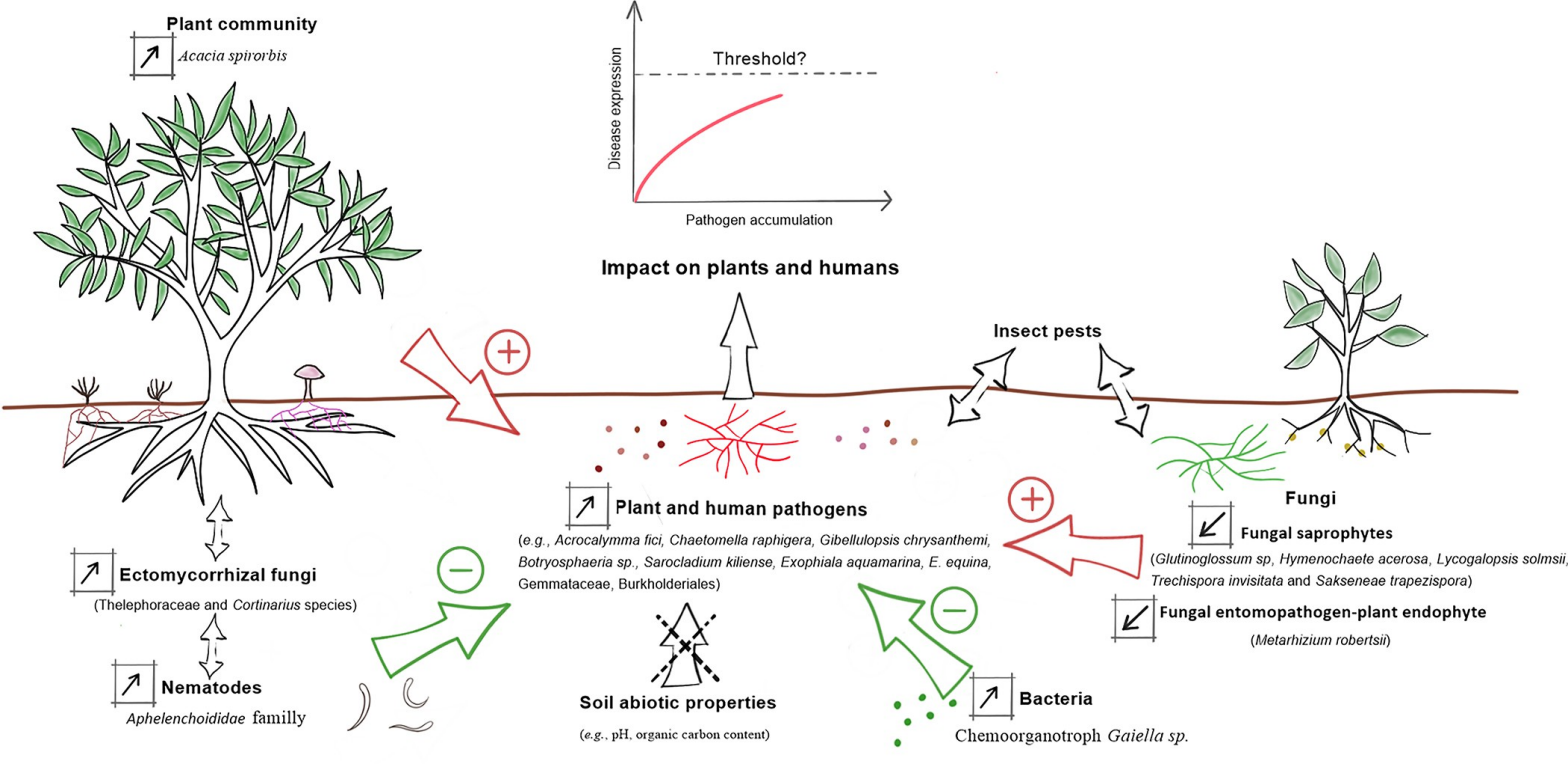
Synthesis of soil environment components potentially involved in the accumulation of plant and human pathogens with the reduction of the fallowing period in traditional yams culture in Maré Island. Detected increases and decreases of each component, via the approaches implemented in *Anaconda*, are represented by arrows within boxes on the left. Related probable positive and negative effects on soil augmentation of detrimental organisms are indicated, *i*.*e*., that may favour (+) or suppress (-) pathogens. Potential interactions between other parameters than pathogens are also shown (between *A*. *spirorbis* and ectomycorrhizal fungi). The absence of apparent abiotic effects is represented. An important aspect also represented is the potentiality of soil pathogen populations reaching a threshold that could lead to substantial plant and human infections.

Conversely, to the reduction of saprophyte and entomopathogen-plant endophyte fungi, an over-representation of the chemoorganotroph *Gaiella* bacterial genus [116] was observed in the short-term fallow via the ‘targeted’ analysis (Fig 6). In tomato cropping soils, after organic amendment, a strong relationship was observed between this genus and the inhibition of the soil pathogen responsible for *Fusarium* wilt [117]. Therefore, in our short fallow system, certain beneficial taxon acting against detrimental soil microorganisms may also be present. The intrinsic balance of soil between its relative abundance of beneficial and detrimental microbes is a crucial factor in determining its capacity to express or suppress diseases. One of the major questions consequently arising is when this threshold leading to one situation or the other would be met (Fig 9).

Other biotic components of the soil environment than fungi and bacteria can contribute to soil suppressiveness [114]. As earlier seen, the fungal pathogenic *Botryosphaeria* genus (Botryosphaeriaceae) was present in both fallows, but particularly in the short one. A *Botryosphaeria* species has been recovered from *Acacia* plant species (Fabaceae) in Australia [118]. According to *Anaconda* results on plant communities, *Acacia spirorbis* was significantly present in both fallows, with higher relative abundances in the short fallow. The larger abundance of this fungus could thus be related to *Acacia*’s abundance. Interestingly, another microorganism type, a nematode of the Aphelenchoididae family has been experimentally demonstrated to feed on a *Botryosphaeriaceae* member [119, 120]. Using again the *Anaconda* package, significant variations in the abundance of this nematode family were detected, with higher abundances in the short fallow. Preferential grazing of an Aphelenchoididae species on ectomycorrhizal fungi has also been revealed in the literature [119]. *A*. *spirorbis* is recognised as an ectomycorrhizal shrub [121], which by the way would explain the over-representation of ectomycorrhizal fungi in the short-term fallowing (*i*.*e*., *Thelephora*ceae and *Cortinarius* species) (Fig 6). Multiple biotic interactions may thus intervene in the regulation of *Botryosphaeria* in soil. This fungus could, as previously indicated, benefit from the larger abundance of *Acacia*, but, at the same time, may be regulated by the predation of nematodes, which are also stimulated by the presence of ectomycorrhizal fungi. *A*. *spirorbis* is also able to form another type of mycorrhiza, *i*.*e*., endomycorrhiza. This characteristic could explain the over-representation in the short fallow, underlined by the ‘global’ analysis (Fig 7), of *Spizellomyces punctatus*, a chytrid species that has been suggested to attack and colonises dead endomycorrhizal spores [122]. *S*. *punctatus* could also be an indicator of perturbation. Lozupone and Klein (2002) [123] showed that *Spizellomyces* populations increased in response to disturbance (*i*.*e*., after experiencing agricultural cultivation); an observation supporting the aforementioned facts that short fallow constitutes a degraded system. The statistical approaches implemented in our *Anaconda* package may, thus, help to disentangle and better understand the multiple biological interactions occurring in a given ecosystem, particularly those leading to an over-representation of certain harmful microbes in soil (Fig 9). It can, additionally, participate in defining ‘targeted’ agricultural management practices to control pathogen populations, for instance, here, by regulating *A*. *spirorbis* occurrence.

Besides biotic factors, abiotic soil properties can also, directly and indirectly (via influencing other soil organisms), be involved in regulating plant and human pathogens populations in soil [7, 124]. Soil attributes, such as pH, soil moisture, organic matter content, and nutrient availability, can have a role in soil pathogen’s establishment, survival and growth [7, 124]. However, in our study, when significant differences occurred (*e*.*g*., pH, organic matter content, carbon content, and C/N ratio), they were mostly between the short fallow and the ecosystem of reference (not with the long fallow). It seems likely that biotic rather than abiotic factors regulate plant and human pathogens in our traditional agricultural system.

## Conclusion

Despite some tendencies, notably in terms of global microbial phyla dominance, ‘classical’ community analysis failed to detect significant changes in microbial diversity, composition, and structure in response to agricultural practices on Maré island. By contrast, our newly developed statistical approaches for community investigation implemented in the *Anaconda* package (i.e., the ‘targeted’ and ‘global’ analyses), clearly revealed differences in the occurrence of soil organisms among the studied systems, especially for fungi. Indeed, a significant over-representation of harmful plant and human fungal pathogens was observed in the short fallow soil. At the same time, an under-representation of beneficial soil microorganisms, such as saprophytic, entomopathogenic and plant-endophyte fungi, was detected. The specific shifts in fungal and bacterial taxa, in combination with the characterisation of other biotic and biotic features, allowed us to infer hypothetical links between these diverse soil environmental components and assume their potential implication in soil pathogen suppression (Fig 9). Our findings undeniably support the major interest in using next-generation sequencing technologies, in combination with more classical ecological inventories, and appropriate statistical methods to establish sensitive, informative and reproducible biological indicators, and subsequently assess disease potential in soils. They also highlight the significance of picking into the omics toolbox by using and transferring, here, methodologies initially developed for genomics-transcriptomics in metabarcoding. In addition to the insights gained from the classical community analysis and the *Anaconda* package, it is important to note that the cultivation of yams holds great cultural and symbolic significance for the local people of Maré Island in New Caledonia. Thus, the impact of changes in agricultural practices on soil health extends beyond the purely ecological and must also be considered within a cultural context. This new tool that is *Anaconda* could further be used for determining the impact in various crop systems of different agricultural practices (e.g., organic amendments and cover crops) on soil microorganisms, and consequently help to find solutions for regulating detrimental microorganisms. Such a combination of ‘targeted’ and ‘global’ analyses could promote the use of eDNA metabarcoding in biomonitoring and represent the next breakthrough in soil health and One Health assessment, as well as in various ecological domains.

## Supporting information

S1 Fig Sampling plan. Five plots of 20 x 20m were established per condition, providing 15 plots in total. In each of the 20 x 20m, four 5 x 5m sub-plots were positioned in the four corners and one in the centre. Within each of these sub-plots, five soil samples were collected at 0–15 cm depth using a five cm diameter auger. All soil samples collected in a given plot were then mixed to form a composite soil sample. Thus, each composite sample corresponds to one plot. A total of 15 composite samples was finally obtained and corresponded to the 15 plots set up in the present work. SF is for Short Fallow; LF is for Long Fallow, and F is for Forest.
(PDF)

S2 Fig Alpha rarefaction plots (observed ASVs, Shannon, and Faith PD) for fungi (ITS2). The alpha rarefaction plots for fungi typically show three curves: observed ASVs, Shannon index, and Faith PD. SF is for Short Fallow; LF is for Long Fallow, and F is for Forest.
(PDF)

S3 Fig Alpha rarefaction plots (observed ASVs, Shannon, and Faith PD) for bacteria (16S). Same legend as the S2 Fig.
(PDF)

S4 Fig Fungi diversity boxplots. The fungi diversity boxplots represent various metrics used to assess the diversity of fungal communities. These metrics include observed ASVs (Amplicon Sequence Variants), Chao1, Simpson, Shannon entropy, Faith PD, Simpson evenness, Pielou evenness, and Fisher alpha. SF is for Short Fallow; LF is for Long Fallow, and F is for Forest.
(PDF)

S5 Fig Bacteria diversity boxplots. Same legend as the S4 Fig.
(PDF)

S6 Fig Soil texture triangle. At the corners of the triangle are three main soil components: sand, silt, and clay. Each dot is a sample that falls within one of the twelve sections.
(PDF)

S7 Fig *Anaconda* R package schema to understand the links between different files and analysis portions. For a better understanding, please refer to the readme document at ‘https://github.com/PLStenger/Anaconda’.
(PDF)

S8 Fig Physico-chemical analysis. Granulometric fraction, physical, linked organic matter, free organic matter, microbial biomass analysis boxplots, microbial biomass, mineralised carbon balance (microbial activity), and mineralised nitrogen balance (microbial activity) analysis boxplot. SF is for Short Fallow; LF is for Long Fallow, and F is for Forest.
(PDF)

S9 Fig db-RDA (distance-based redundancy analysis) plot of the fungal phyla in relation to the granulometric fractions (clay, silt, and sand; in per cent) of soil samples collected from three different land-use types: Short fallow (SF), long fallow (LF), and forest (F). The plot displays the distribution of the fungal phyla in relation to the granulometric fractions of the soil samples, with each point representing a sample.
(PDF)

S10 Fig Dispersion (A and C) and sparsity (B and D) plot for fungi (A and B) and bacteria (C and D). Dispersion and sparsity plots are used to assess the data quality and the statistical model’s appropriateness. A dispersion plot shows the relationship between the mean of normalised counts and their variance (or dispersion) for each ASV. The dispersion estimates are calculated using a negative binomial model, and the plot is typically shown on a logarithmic scale to visualise the trend. A good dispersion plot shows a relatively constant dispersion across all normalised count levels, which indicates that the negative binomial model is appropriate for the data. A sparsity plot shows the proportion of ASVs with a given number of counts in the sample. It is used to assess the overall level of sequencing depth and the quality of the normalisation procedure. The plot typically shows a decreasing trend, with the majority of ASVs having low counts and a smaller proportion having higher counts. If the sparsity plot shows a high proportion of ASVs with low counts, it suggests that the sequencing depth is insufficient, or the normalisation procedure is inadequate. In contrast, if the sparsity plot shows a high proportion of ASVs with very high counts, it may indicate a technical artefact or batch effect that needs to be addressed.
(PDF)

S11 Fig Pheatmap log2 norm counts with taxonomy for fungi from the *Anaconda* R package. The heatmap displays the relative abundance of the 75 most abundant fungal Amplicon Sequence Variants (ASVs) across multiple samples. The log2 normalised counts of each ASV were used to generate the heatmap, which allows for the comparison of relative abundance between different ASVs and samples. The heatmap also includes taxonomic information for each ASV, which allows for the identification of taxonomic groups that are more abundant in certain samples or conditions. The heatmap is clustered based on the Euclidean distance between samples and ASVs using the average clustering method, which groups samples and ASVs with similar abundance patterns together. This allows for the identification of clusters of samples or ASVs that share similar characteristics or respond similarly to certain conditions. SF is for Short Fallow; LF is for Long Fallow, and F is for Forest.
(PDF)

S12 Fig Pheatmap log2 norm counts with taxonomy for bacteria from the *Anaconda* R package. Same legend as the S11 Fig.
(PDF)

S1 Table MultiQC results for fungi. Total number of sequences and their means (and standard deviation) by condition (SF is for Short Fallow; LF is for Long Fallow, and F is for Forest), before and after the Trimmomatic step, percentage of kept sequences.
(XLSX)

S2 Table QIIME2 stats for fungi. Total number of sequences and their means (and standard deviation) by condition (SF is for Short Fallow; LF is for Long Fallow, and F is for Forest) for each QIIME2 step (input, filtered, percentage of input passed filter, denoised, merged, percentage of input merged, mean, SD, non-chimeric, percentage of input non-chimeric, mean, SD, Table, ConTable, and Rarefaction).
(XLSX)

S3 Table MultiQC results for bacteria. Same legend as the S1 Table.
(XLSX)

S4 Table QIIME2 stats for bacteria. Same legend as the S2 Table.
(XLSX)

S5 Table Organophysico-chemicals analysis. SF is for Short Fallow; LF is for Long Fallow, and F is for Forest.
(XLSX)

S6 Table Plantae statistics results for the 29 found plant species. SF is for Short Fallow; LF is for Long Fallow, and F is for Forest.
(XLSX)

S7 Table Nematoda statistics results for the 36 found nematoda families. SF is for Short Fallow; LF is for Long Fallow, and F is for Forest.
(XLSX)
