# Supplementary figures and images for: Advancing biomonitoring of eDNA studies with the Anaconda R package: Integrating soil and One Health perspectives in the face of evolving traditional agriculture practices
Source: PLoS One. 2025 Jan 16;20(1):e0311986. doi: 10.1371/journal.pone.0311986 (PMC11737689; doi:10.1371/journal.pone.0311986)

alpha-rarefaction observed ASVs

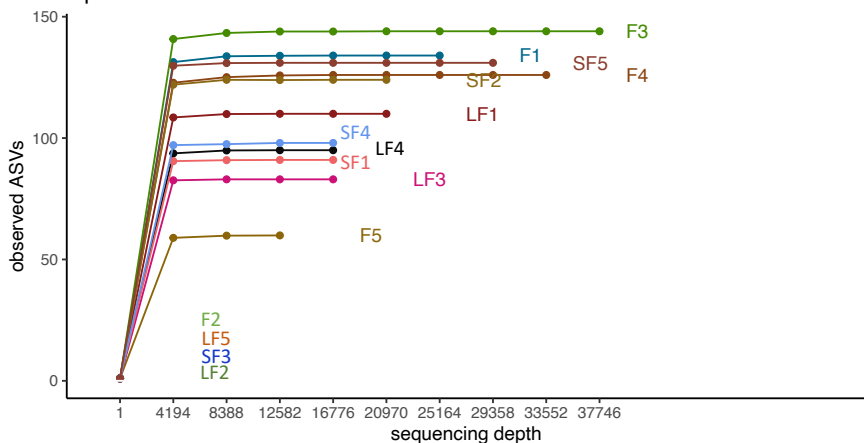

alpha-rarefaction Shannon

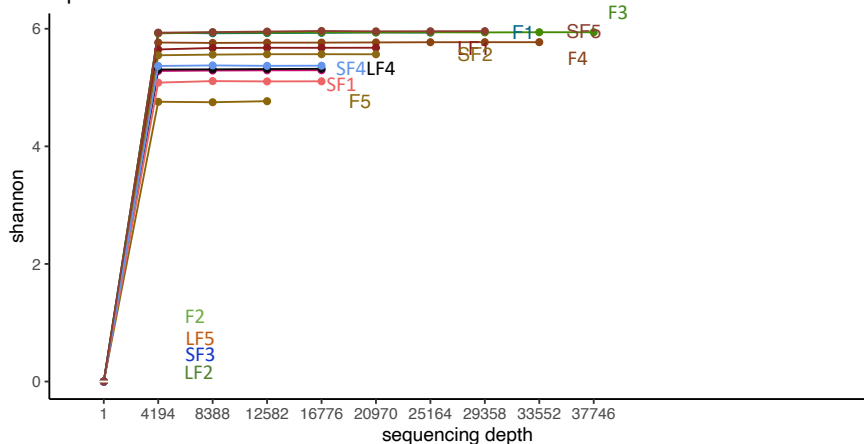

alpha-rarefaction faith\_pd

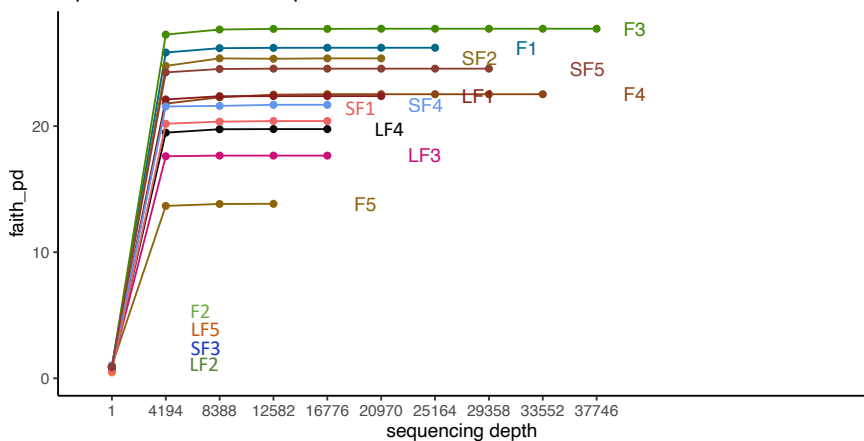

Supplement: S2 Fig — The alpha rarefaction plots for fungi typically show three curves: observed ASVs, Shannon index, and Faith PD. SF is for Short Fallow; LF is for Long Fallow, and F is for Forest. (PDF) [file pone.0311986.s002.pdf]

alpha-rarefaction observed ASVs

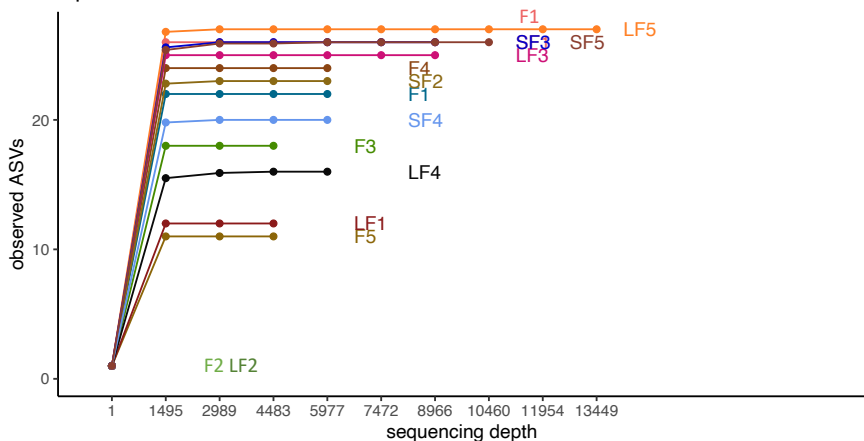

alpha-rarefaction Shannon

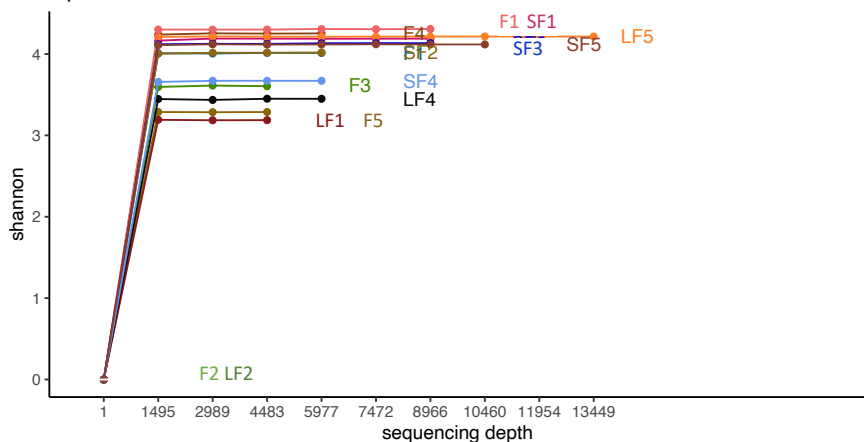

alpha-rarefaction faith\_pd

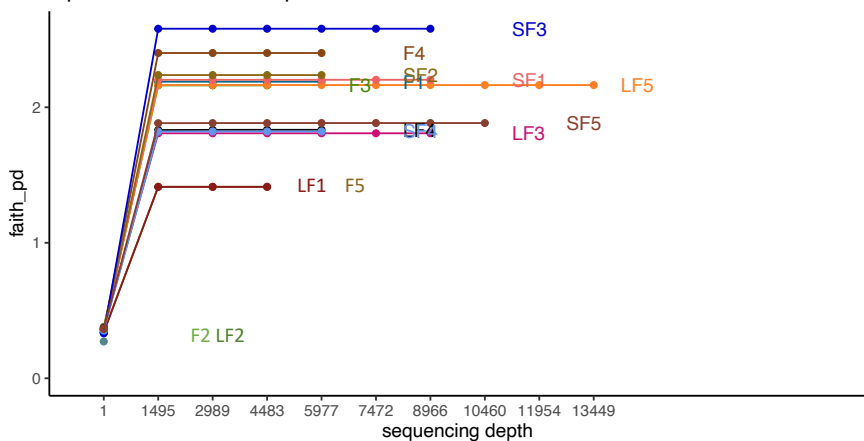

Supplement: S3 Fig — Same legend as the S2 Fig. (PDF) [file pone.0311986.s003.pdf]

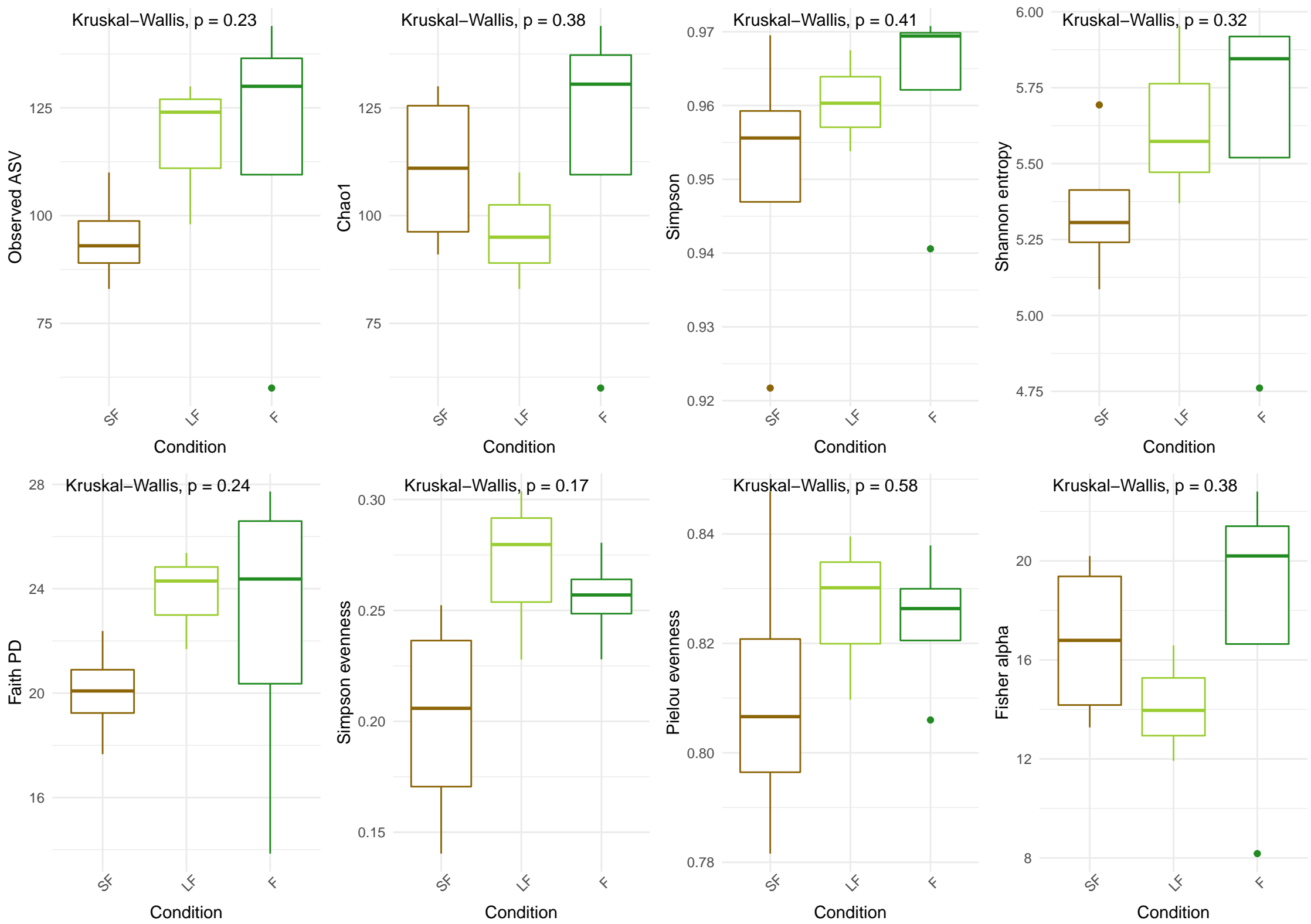

Supplement: S4 Fig — The fungi diversity boxplots represent various metrics used to assess the diversity of fungal communities. These metrics include observed ASVs (Amplicon Sequence Variants), Chao1, Simpson, Shannon entropy, Faith PD, Simpson evenness, Pielou evenness, and Fisher alpha. SF is for Short Fallow; LF is for Long Fallow, and F is for Forest. (PDF) [file pone.0311986.s004.pdf]

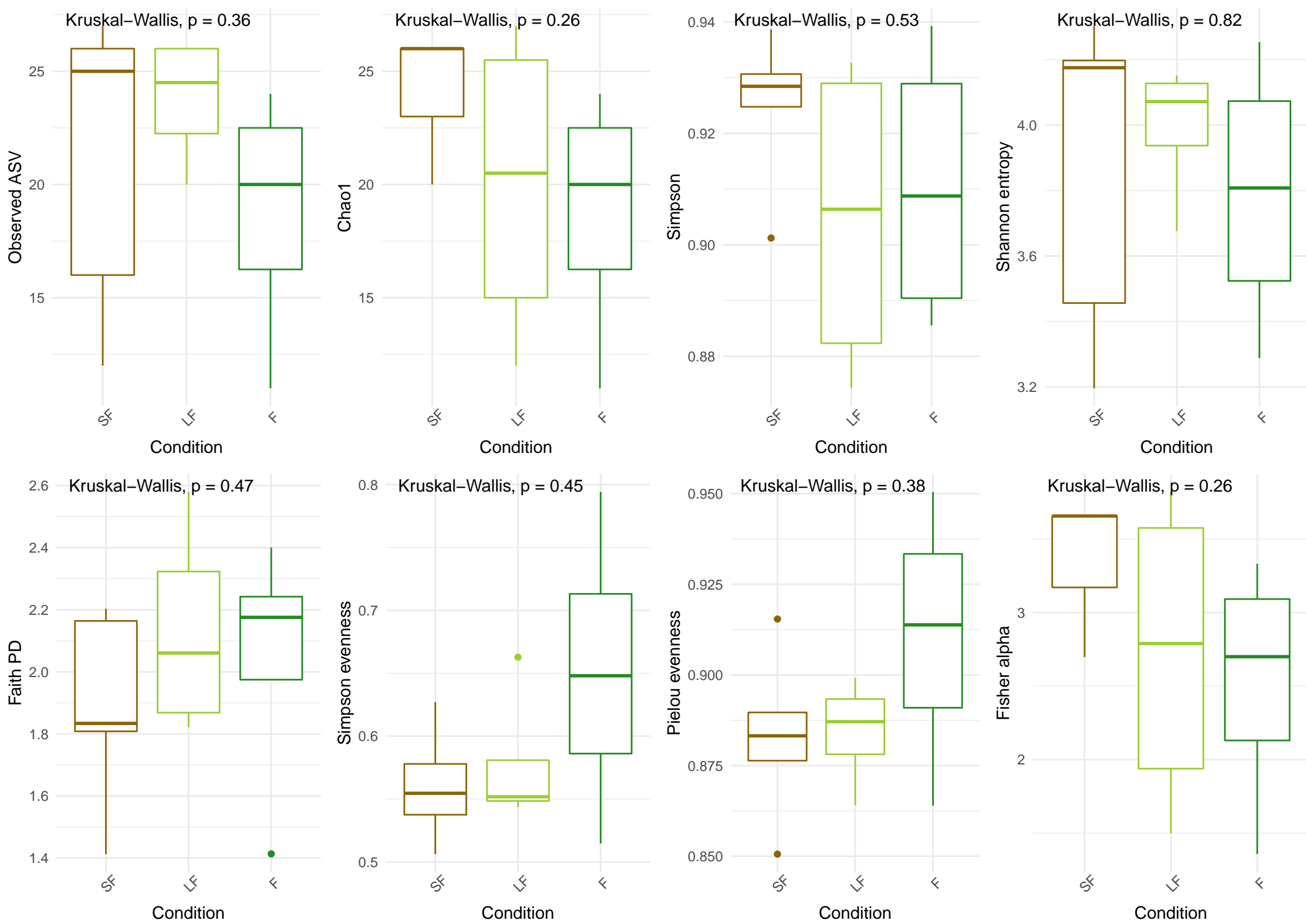

Supplement: S5 Fig — Same legend as the S4 Fig. (PDF) [file pone.0311986.s005.pdf]

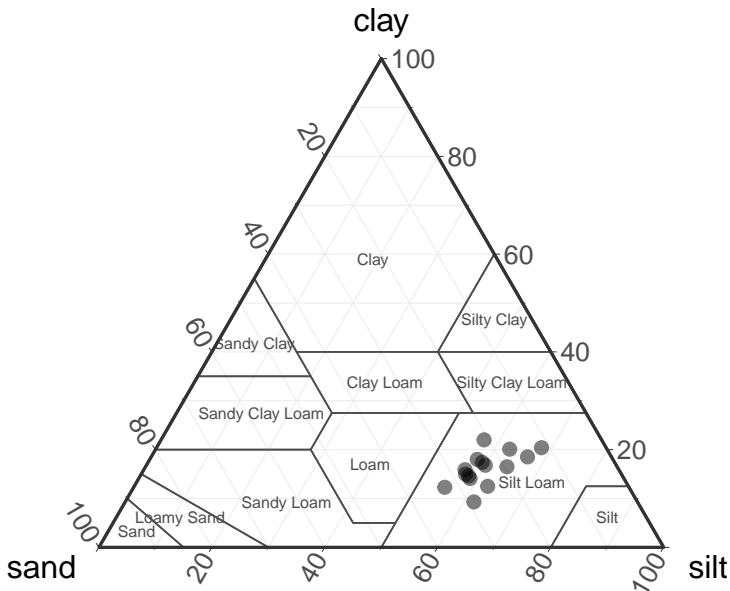

Supplement: S6 Fig — At the corners of the triangle are three main soil components: sand, silt, and clay. Each dot is a sample that falls within one of the twelve sections. (PDF) [file pone.0311986.s006.pdf]

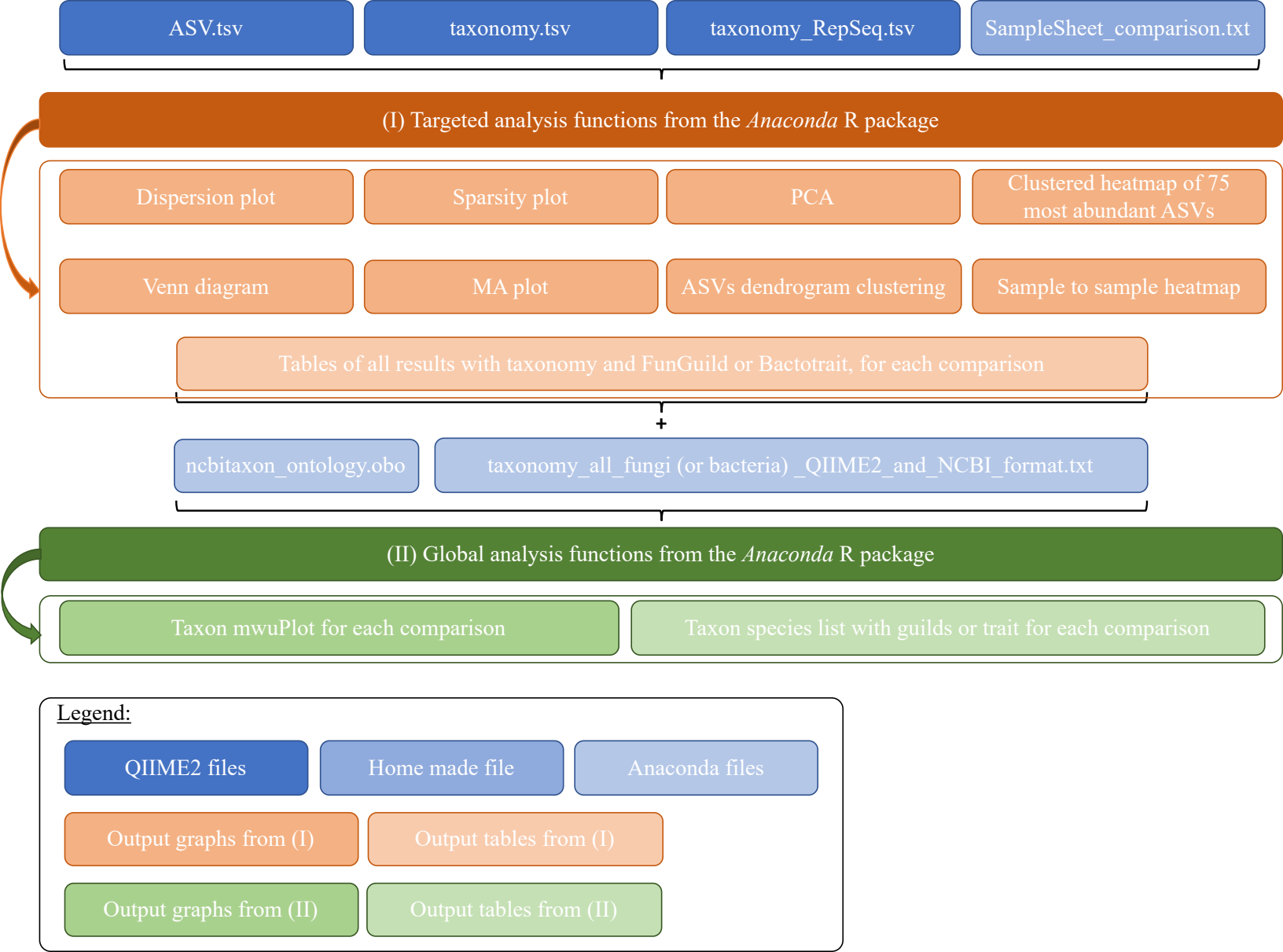

Supplement: S7 Fig — For a better understanding, please refer to the readme document at ‘https://github.com/PLStenger/Anaconda’. (PDF) [file pone.0311986.s007.pdf]

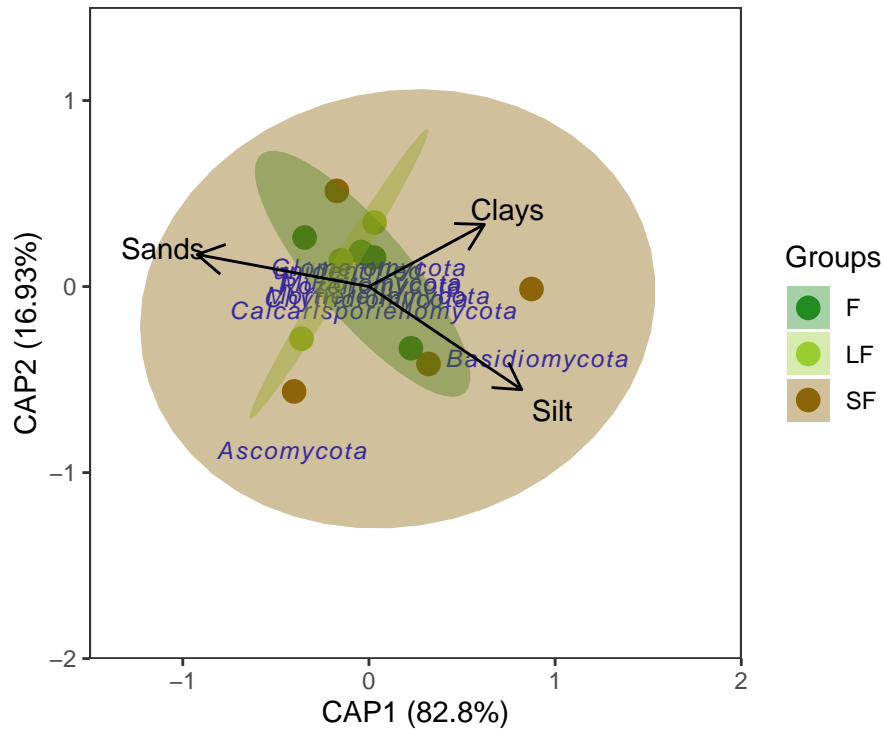

Supplement: S9 Fig — The plot displays the distribution of the fungal phyla in relation to the granulometric fractions of the soil samples, with each point representing a sample. (PDF) [file pone.0311986.s009.pdf]

A

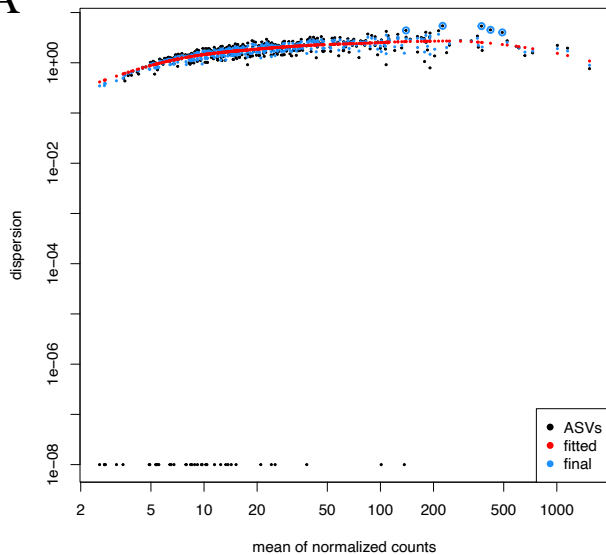

B

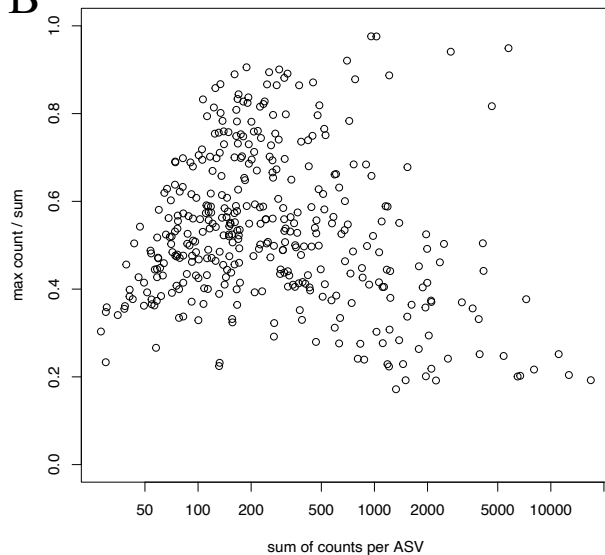

C

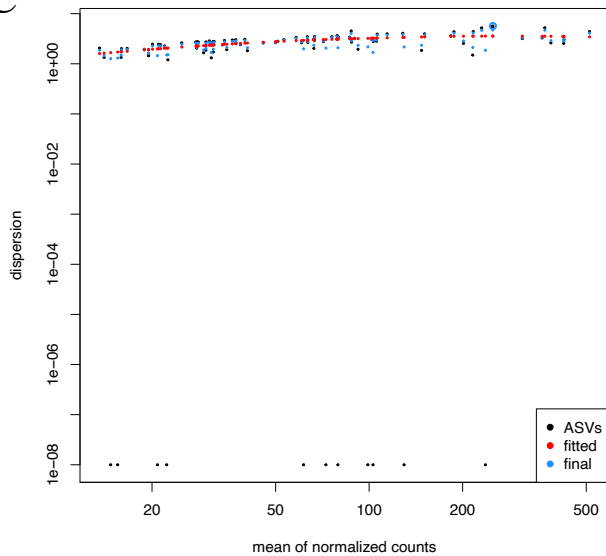

D

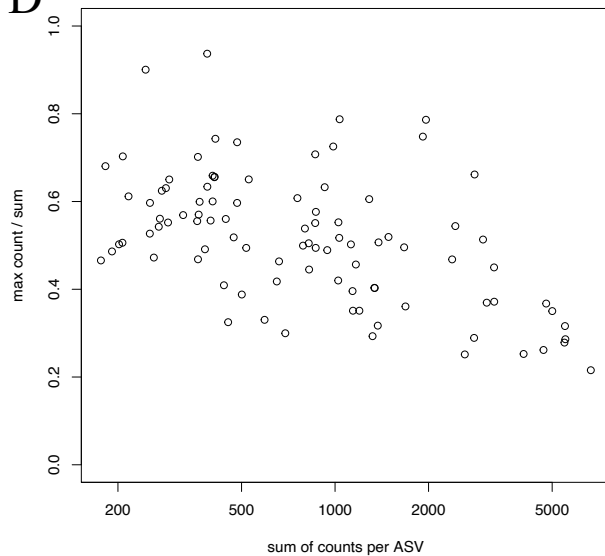

Supplement: S10 Fig — Dispersion (A and C) and sparsity (B and D) plot for fungi (A and B) and bacteria (C and D). Dispersion and sparsity plots are used to assess the data quality and the statistical model’s appropriateness. A dispersion plot shows the relationship between the mean of normalised counts and their variance (or dispersion) for each ASV. The dispersion estimates are calculated using a negative binomial model, and the plot is typically shown on a logarithmic scale to visualise the trend. A good dispersion plot shows a relatively constant dispersion across all normalised count levels, which indicates that the negative binomial model is appropriate for the data. A sparsity plot shows the proportion of ASVs with a given number of counts in the sample. It is used to assess the overall level of sequencing depth and the quality of the normalisation procedure. The plot typically shows a decreasing trend, with the majority of ASVs having low counts and a smaller proportion having higher counts. If the sparsity plot shows a high proportion of ASVs with low counts, it suggests that the sequencing depth is insufficient, or the normalisation procedure is inadequate. In contrast, if the sparsity plot shows a high proportion of ASVs with very high counts, it may indicate a technical artefact or batch effect that needs to be addressed. (PDF) [file pone.0311986.s010.pdf]

Clustered heatmap of 75 most abundant ASVs

euclidean distance with average clustering method

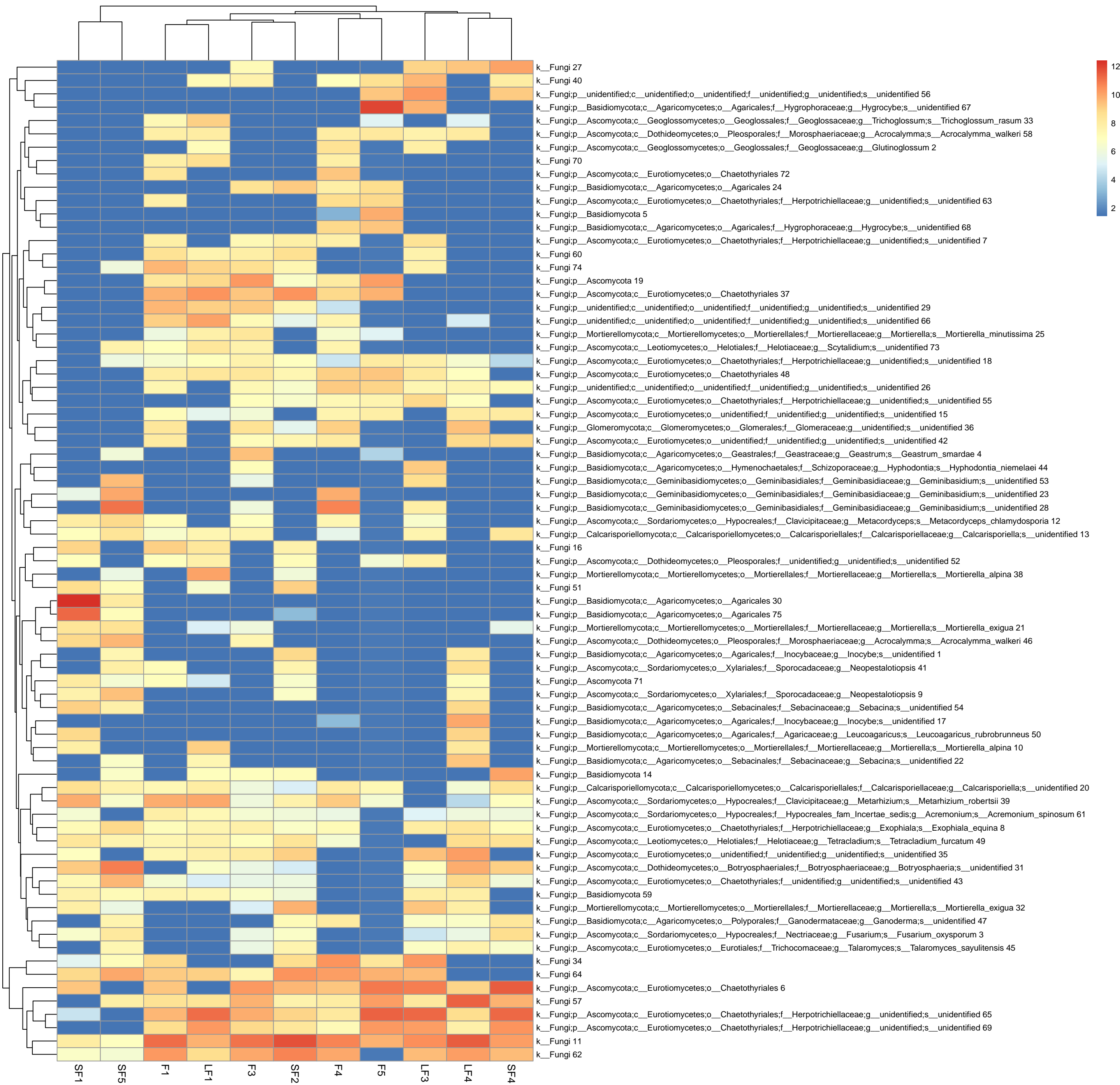

Supplement: S11 Fig — The heatmap displays the relative abundance of the 75 most abundant fungal Amplicon Sequence Variants (ASVs) across multiple samples. The log2 normalised counts of each ASV were used to generate the heatmap, which allows for the comparison of relative abundance between different ASVs and samples. The heatmap also includes taxonomic information for each ASV, which allows for the identification of taxonomic groups that are more abundant in certain samples or conditions. The heatmap is clustered based on the Euclidean distance between samples and ASVs using the average clustering method, which groups samples and ASVs with similar abundance patterns together. This allows for the identification of clusters of samples or ASVs that share similar characteristics or respond similarly to certain conditions. SF is for Short Fallow; LF is for Long Fallow, and F is for Forest. (PDF) [file pone.0311986.s011.pdf]

Clustered heatmap of 75 most abundant ASVs  
euclidean distance with average clustering method

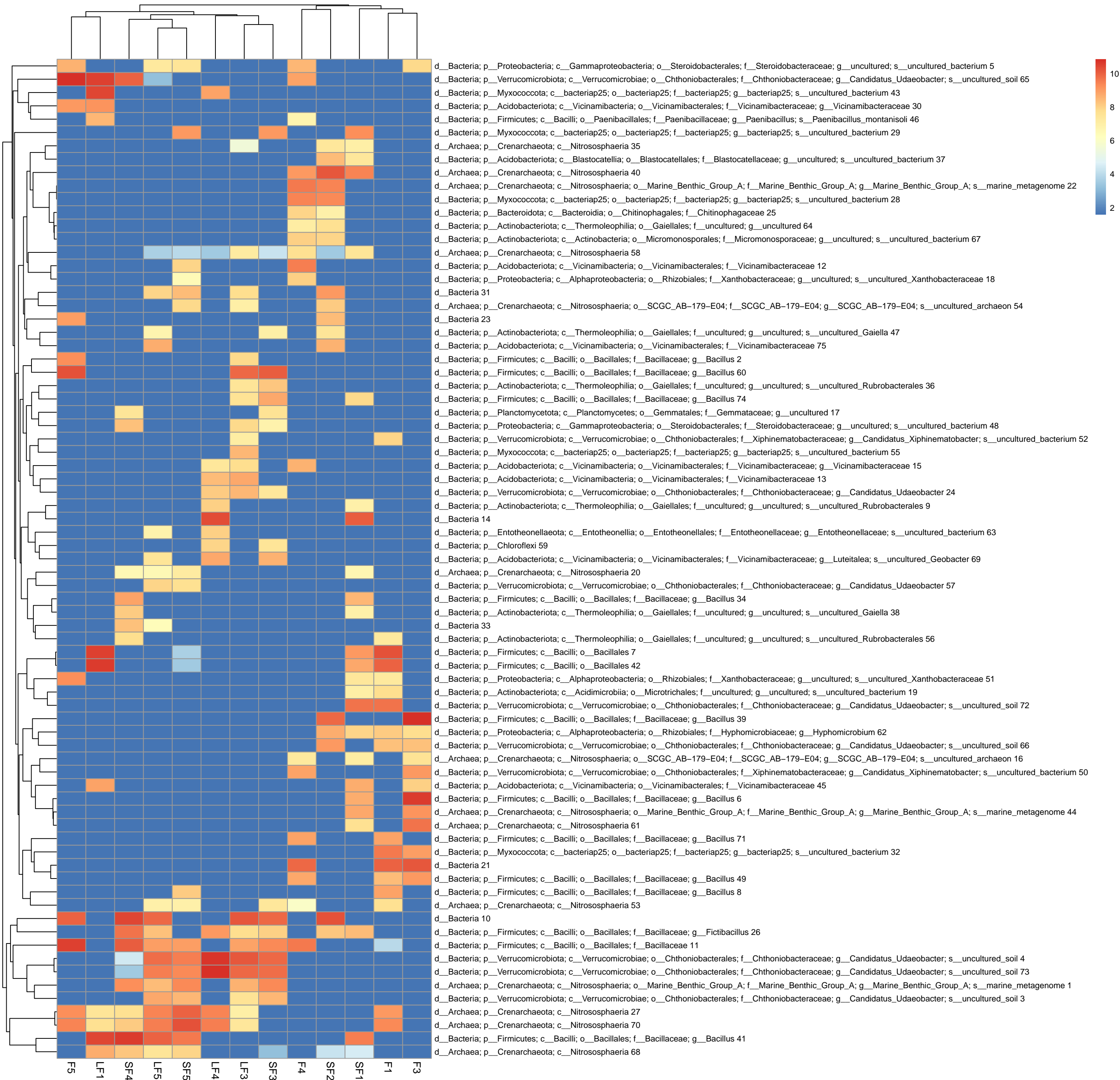

Supplement: S12 Fig — Same legend as the S11 Fig. (PDF) [file pone.0311986.s012.pdf]
