# Supplementary material for: Advancing biomonitoring of eDNA studies with the Anaconda R package: Integrating soil and One Health perspectives in the face of evolving traditional agriculture practices
Source: PLoS One. 2025 Jan 16;20(1):e0311986. doi: 10.1371/journal.pone.0311986 (PMC11737689; doi:10.1371/journal.pone.0311986)

Granulometric fraction

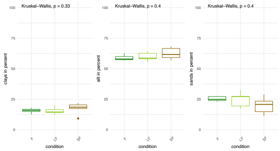

Physical analysis

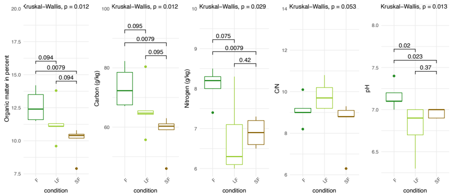

Linked Organic Matter

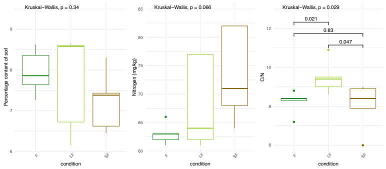

Free Organic Matter

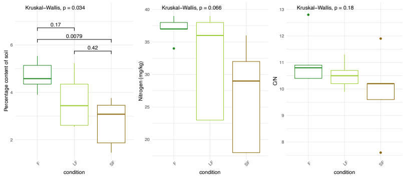

Microbial biomass

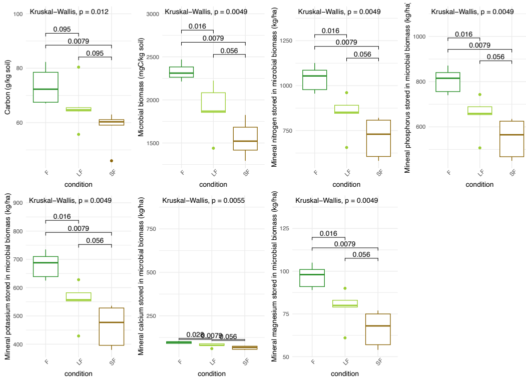

Mineralized Carbon Balance (Microbial Activity)

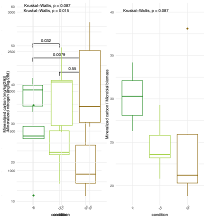

Supplement: S8 Fig — Granulometric fraction, physical, linked organic matter, free organic matter, microbial biomass analysis boxplots, microbial biomass, mineralised carbon balance (microbial activity), and mineralised nitrogen balance (microbial activity) analysis boxplot. SF is for Short Fallow; LF is for Long Fallow, and F is for Forest. (PDF) [file pone.0311986.s008.pdf]
