# Supplementary material for: Advancing biomonitoring of eDNA studies with the Anaconda R package: Integrating soil and One Health perspectives in the face of evolving traditional agriculture practices
Source: PLoS One. 2025 Jan 16;20(1):e0311986. doi: 10.1371/journal.pone.0311986 (PMC11737689; doi:10.1371/journal.pone.0311986)
Supplement: S1 Fig — Five plots of 20 x 20m were established per condition, providing 15 plots in total. In each of the 20 x 20m, four 5 x 5m sub-plots were positioned in the four corners and one in the centre. Within each of these sub-plots, five soil samples were collected at 0–15 cm depth using a five cm diameter auger. All soil samples collected in a given plot were then mixed to form a composite soil sample. Thus, each composite sample corresponds to one plot. A total of 15 composite samples was finally obtained and corresponded to the 15 plots set up in the present work. SF is for Short Fallow; LF is for Long Fallow, and F is for Forest. (PDF) [file pone.0311986.s001.pdf]

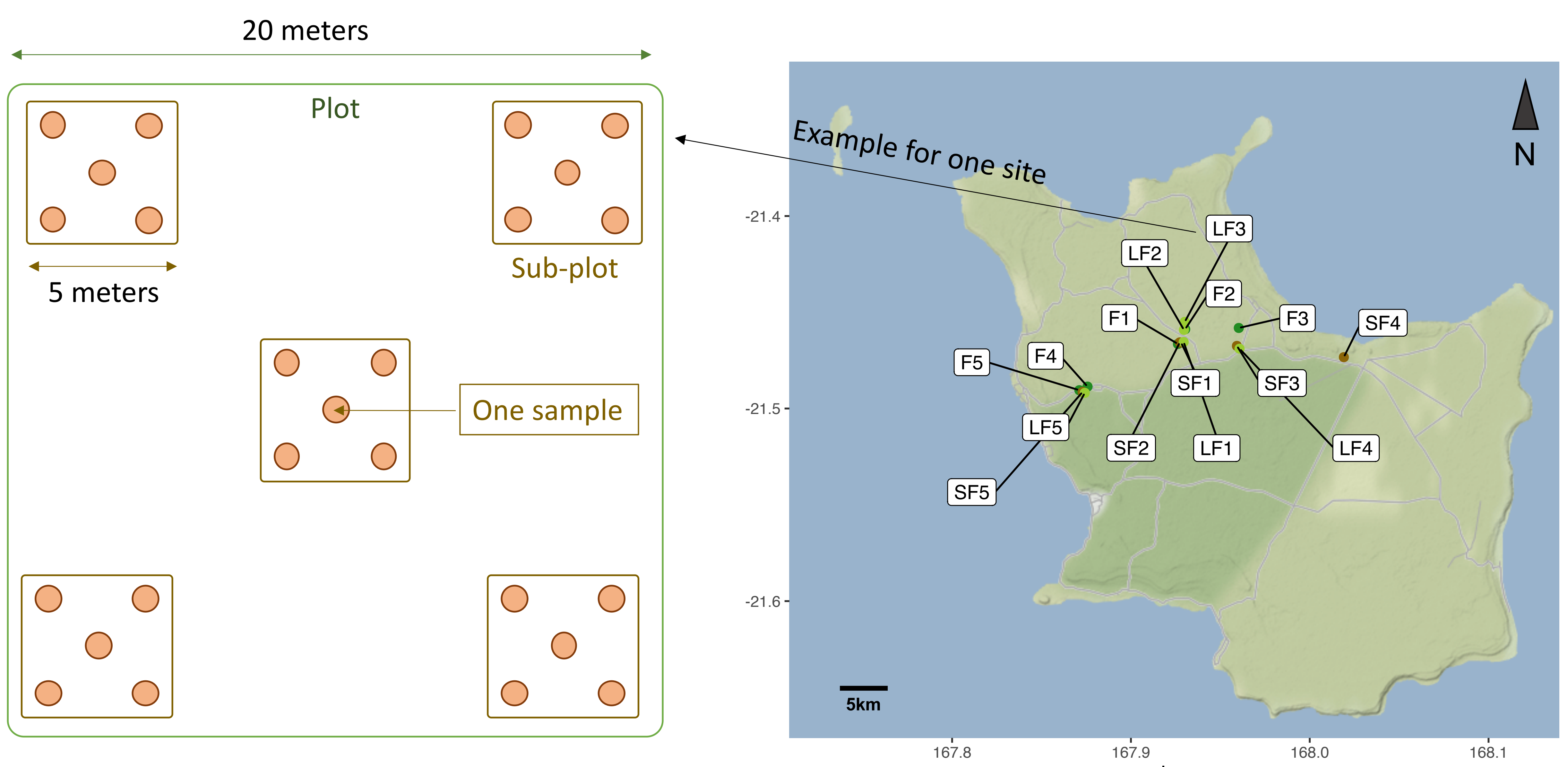

20 meters
Plot
Sub-plot
5 meters
One sample
Example for one site
N
LF3
LF2
F2
F1
F3
SF4
F4
F5
SF1
SF3
LF5
SF2
LF1
LF4
SF5
5km
-21.4
-21.5
-21.6
167.8
167.9
168.0
168.1
